# Design and Synthesis of Polyamine‐Proteolysis Targeting Chimera Conjugates for Histone Deacetylase (HDAC) Degradation with Enhanced Cellular Uptake

**DOI:** 10.1002/open.202500356

**Published:** 2025-11-02

**Authors:** Yanran Liu, Wentian Chen, Yanwei Shang, Chaonan Tang, Xianming Zeng, Jun Li, Wenting Du

**Affiliations:** ^1^ Hangzhou Medical College Hangzhou 311399 P. R. China

**Keywords:** histone deacetylase, polyamine transport, polyamine, proteolysis targeting chimera, Vorinostat

## Abstract

Although histone deacetylase (HDAC) inhibitors have demonstrated significant advantages in the field of targeted cancer therapy, numerous adverse events have been observed due to the high doses required to achieve therapeutic effects. Additionally, acquired drug resistance to HDAC inhibitors has also been observed in clinical usage. Given these findings, the development of HDAC degraders may represent a more promising strategy to overcome these limitations due to their specific mechanism of action. In this study, 14 HDAC degraders featuring a polyamine linker are designed and synthesized by conjugating HDAC inhibitors (HDACi, Vorinostat) with Cereblon (CRBN, an E3 ubiquitin ligase ligand). Significantly, compound **I** exhibited a degradation efficiency of ≈62% at 5 μM in MDA‐MB‐231 cells. Additionally, compound **N** exhibited the highest cellular uptake efficiency in a dose‐ and time‐dependent manner. The findings presented in our manuscript provided valuable insights for the development of a proteolysis targeting chimera with high cellular uptake efficiency.

## Graphical abstract

In this article, proteolysis targeting chimeras (PROTACs) bearing a polyamine linker capable of crossing cancer cell membranes via the polyamine transport system are synthesized and evaluated. The results provide valuable insights for the design of PROTACs with enhanced cellular uptake efficiency.

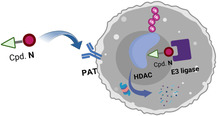

## 1 Introduction

Histone deacetylases (HDACs), as critical epigenetic regulators, play a pivotal role in essential biological processes, including cell differentiation, proliferation, migration, and apoptosis, by catalyzing the removal of acetyl groups from the side chains of acetylated lysine residues in histones and other protein substrates.^[^ ^1^ ^]^ An increasing number of studies have been identified that HDACs’ disorder could result in related diseases.^[^ ^2^ ^]^ More notably, the overexpression of HDACs has been consistently observed in various types of tumors, with a particularly strong association in breast cancer.^[^ ^3^, ^4^ ^–^ ^5^ ^]^ In breast cancer, this epigenetic silencing of genes involved in cell cycle control, apoptosis, and DNA repair promotes tumor growth and progression. Additionally, HDACs regulate the expression of genes involved in epithelial‐mesenchymal transition, a process critical for cancer cell invasion and metastasis.^[^ ^6^, ^7^ ^–^ ^8^ ^]^ Given their critical role in the progression of breast cancer, HDACs have emerged as a promising therapeutic target for the treatment of this malignancy.^[^ ^9^ ^]^

**Figure 1:**
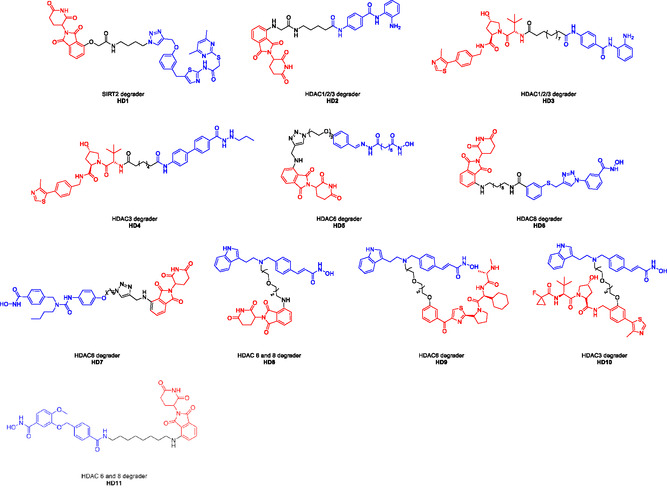
The representative HDAC‐PROTACs.

To date, a range of HDAC inhibitors (HDACi) have been developed, with several receiving Food and Drug Administration (FDA) approval for use in related cancer therapies.^[^ ^10^ ^]^ Unfortunately, challenges such as acquired drug resistance, the need for higher drug doses, and intolerable cytotoxicity have restricted their broader clinical application.^[^ ^11^, ^12^ ^–^ ^13^ ^]^ Proteolysis targeting chimera (PROTAC) technology, which selectively degrades proteins of interest (POI) via the ubiquitin‐proteasome system, presents a novel therapeutic strategy to address the limitations mentioned above.^[^ ^14^ ^]^ Recently, HDAC‐PROTACs have seen significant development following the first HDAC degrader SIRT‐2 in 2017.^[^ ^15^ ^]^ To date, some HDAC‐PROTACs have been successfully designed and synthesized for the treatment of corresponding disorders (**Figure** **1**).^[^ ^16^ ^]^ Compared to traditional HDACis, HDAC‐PROTACs demonstrate superior selectivity, enhanced antiproliferative efficacy, and the ability to overcome drug resistance.^[^ ^17^ ^]^ However, its broad applicability has been hindered by poor cell permeability and limited cellular uptake because of its unsatisfactory drugability.^[^ ^18^ ^]^ Therefore, enhancing cell permeability and cellular uptake is crucial for the development of HDAC‐PROTACs.

Polyamines are aliphatic nitrogen‐containing compounds featuring two or more amino groups, ubiquitously present in both prokaryotic and eukaryotic organisms, where they play vital roles in various cellular processes.^[^ ^19^ ^]^ Notably, cancer cells exhibit heightened uptake of exogenous polyamines through the polyamine transport system (PTS) to sustain their rapid proliferation compared to normal cells.^[^ ^20^ ^]^ More importantly, PTS in cancer cells exhibits structural flexibility, enabling it to accommodate a diverse range of polyamine structures.^[^ ^21^ ^]^ For instance, some cytotoxic‐polyamine conjugates have been developed as antitumor agents utilizing the PTS.^[^ ^22^, ^23^, ^24^ ^–^ ^25^ ^]^ In summary, the use of polyamine conjugates represents a highly promising strategy for the improvement of cell permeability and cellular uptake. However, to the best of our knowledge, the application of polyamine conjugates in HDAC‐PROTACs has not yet been reported. In this article, we induced the polyamine‐conjugate strategy to design and synthesize a series of HDAC‐PROTACs. These degraders not only displayed an enhanced profile of cell permeability and cellular uptake but also exhibited acceptable degradation activity. These findings offer valuable insights and guidance for the development of PROTAC.

## 2 Results and Discussion

### 2.1 Design and Synthesis of a Small HDAC‐PROTAC Library

We designed and synthesized 14 degraders by conjugating the pan‐HDAC inhibitor Vorinostat with CRBN E3 ligands. Our goal was to explore how the length and composition of the PROTAC linker influence their efficacy in inducing HDAC degradation and enabling targeted drug delivery. The PROTACs were synthesized using alkyl linkers, nitrogen‐containing alkyl linkers, and polyethylene glycol (PEG) linkers, with lengths ranging from 3 to 11 atoms.

**Scheme 1:**
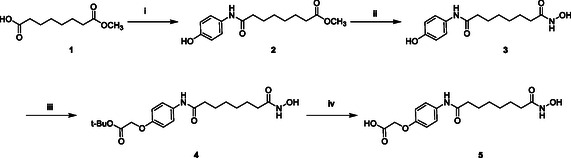
Synthesis of Compounds **5**. The reagents and conditions were as follows: i) 4‐aminophenol, HATU, DIPEA, 15 °C, 5 h; ii) 50% NH_2_OH, MeOH, 60 °C, 12 h; iii) *tert*‐butyl bromoacetate, K_2_CO_3_, DMF, 20 °C, 12 h; and iv) TFA, DCM, 15 °C.

The synthesis of POI ligand **5** is outlined in **Scheme** **1**. Initially, compound **2** was synthesized by a common condensation reaction. Subsequently, intermediate **2** underwent a series of transformations, including aminohydroxylation, *tert*‐butylation, and carboxylation, to yield the final POI ligand **5**.

**Scheme 2:**
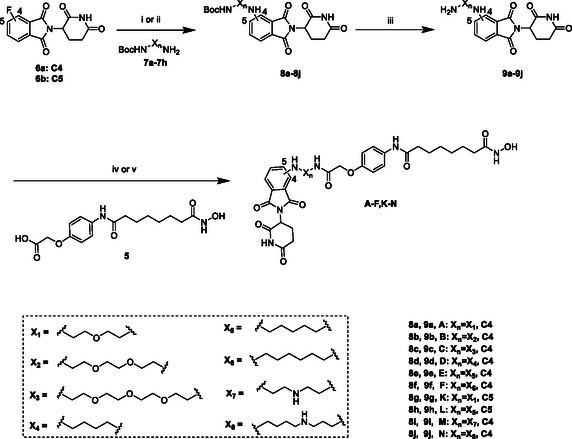
Synthesis of compounds **A–F, K, L, M, N**. The reagents and conditions were as follows: i) DIPEA, NMP, 25 °C, 12 h; ii) DIPEA, DMSO, 25 °C, 6 h; iii) TFA, DCM, 25 °C, 4 h; iv) EDCI, HOAT, NMM, DMSO, 25°C, 12 h; and v) HATU, DIPEA, DMF, 25°C, 12 h.

The synthesis route of compounds **A–F, K, L, M**, and **N** is detailed in **Scheme** **2**. Pomalidomide analogs **8a–j** were prepared via an S_N_Ar reaction between racemic fluorothalidomide and N‐Boc‐protected linkers. The resulting intermediates 8a‐j were then deprotected under acidic conditions to yield the corresponding amine intermediates **9a–j**. Finally, **A–F, K, L, M**, and **N** were obtained by condensation reaction with the ligand **5**.

**Scheme 3:**
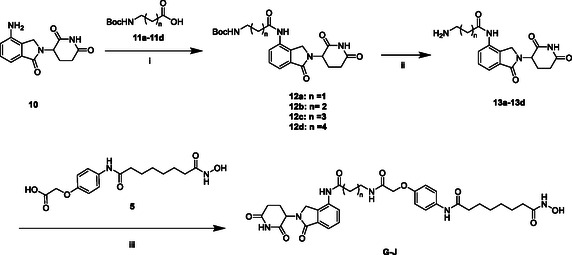
Synthesis of compounds **G–J**. The reagents and conditions were as follows: i) EDCI, HOAT, NMM, DMSO, 25 °C, 12 h; ii) TFA, DCM, 25 °C, 4 h; and iii) HATU, DIPEA, DMF, 25 °C, 12 h.

The synthesis of compounds **G–J** is outlined in **Scheme** **3**. Lenalidomide analogs **12a–d** were synthesized by reacting lenalidomide **10** with N‐Boc‐protected amino acids **11a–d**. The resulting intermediates **12a–d** were then deprotected to yield the corresponding amine intermediates **13a–d**. Finally, compounds **G–J** were obtained by coupling intermediates **13a–d** with **5** using a common condensation reaction.

**Scheme 4:**
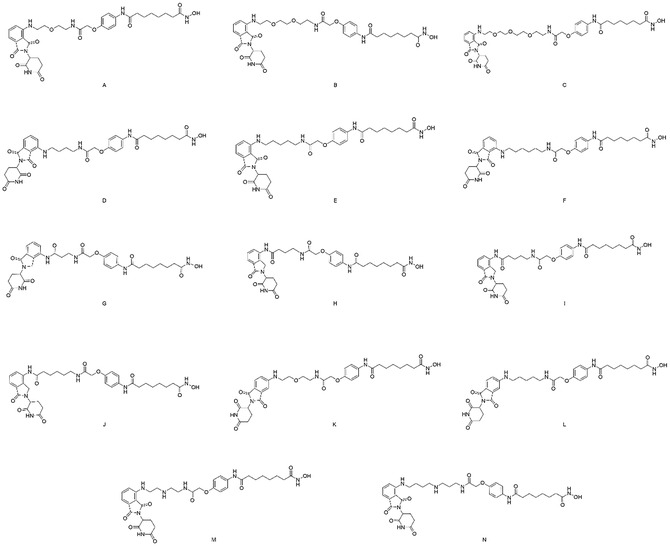
The synthesized HDAC‐PROTACs with different linker structures.

Finally, PROTACs **A–N** with varying linker lengths were obtained as demonstrated in **Scheme** **4**. Main text paragraph.

### 2.2 Characterization of the Degradative Activity of A–N

**Figure 2:**
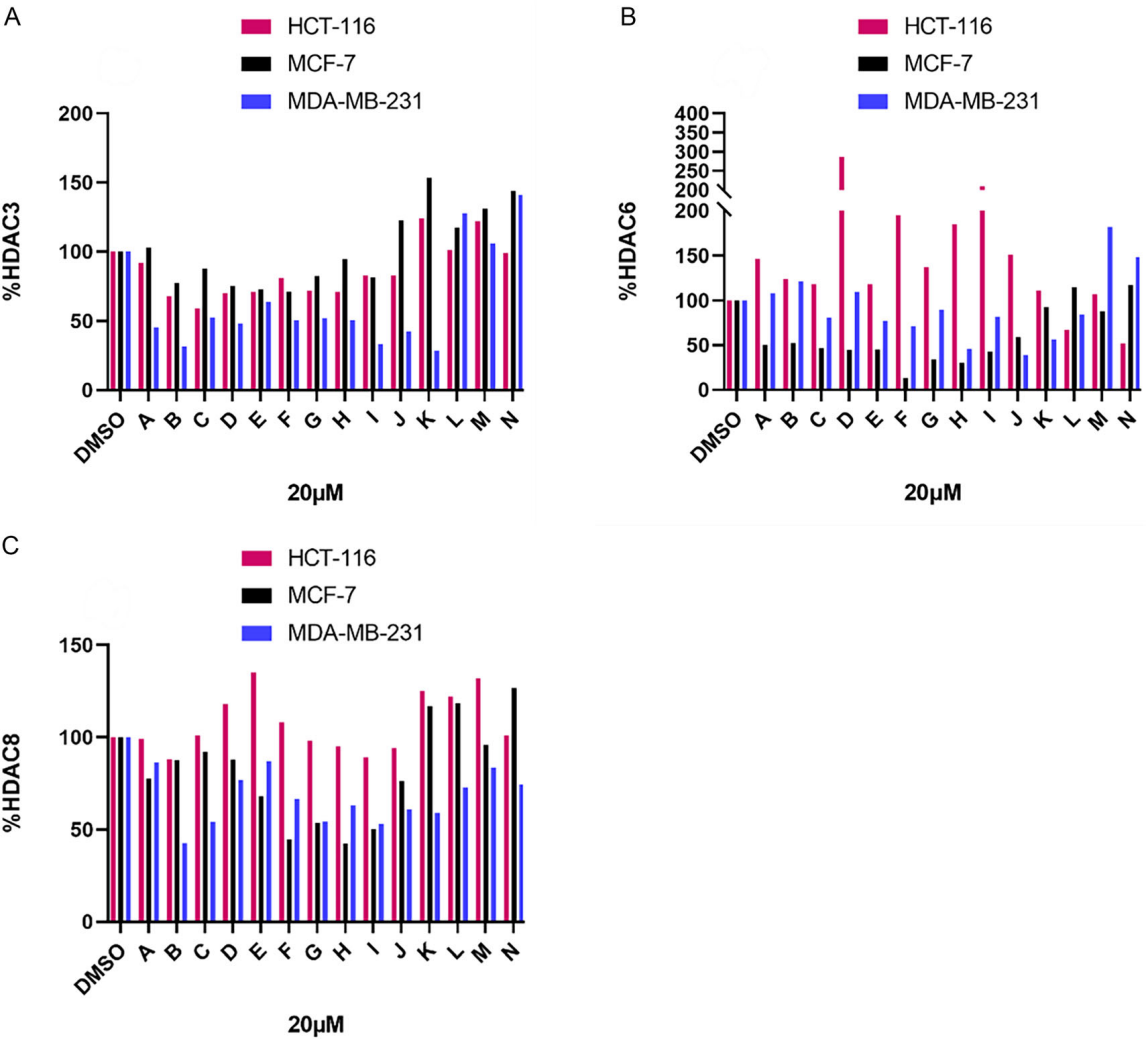
A) Compounds **A–N** were screened at 20 μM with HDAC3 abundance determined by quantitative western blotting in HCT‐116, MCF‐7, and MDA‐MB‐231 cells; B) Compounds **A–N** were screened at 20 μM with HDAC6 abundance determined by quantitative western blotting in HCT‐116, MCF‐7, and MDA‐MB‐231 cells; C) Compounds **A‐N** were screened at 20 μM with HDAC8 abundance determined by quantitative western blotting in HCT‐116, MCF‐7, and MDA‐MB‐231 cells.

Following the synthesis, the degradation activity of the target compounds was evaluated. In our initial investigation, we conducted a preliminary screening to evaluate the degradation activity targeting HDAC3/6/8 in the presence of putative degraders featuring varying linker lengths and compositions. These included PEG‐based linkers (**A, B, C, K)**, alkane‐based linkers (**D, E, F, L**), carboxylic acid‐based linkers (**G, H, I, J**), and polyamine‐based linkers **(M, N)**. We evaluated the effects of these putative HDAC degraders at a concentration of 20 μM in HCT‐116, MCF‐7, and MDA‐MB‐231 cells over a 24 h (western blots available in Figure S1, Supporting Information). Notably, variations at the linker demonstrated a significant impact on the degradation activity. Notably, in MCF‐7 and MDA‐MB‐231 cells, a 6‐atom chain demonstrated significantly stronger HDAC6 degradation activity (**Figure** **2**A–F, **J**), whereas incorporating a −CH_2_OCH_2_− moiety in the linker enhanced HDAC3 degradation (Figure 2B, **K**). Treatment with compounds **A‐N** (20 μM) showed that most were less potent in HCT‐116 cells than in MCF‐7 and MDA‐MB‐231 cells at inducing HDAC6 and HDAC8 protein degradation.

**Figure 3:**
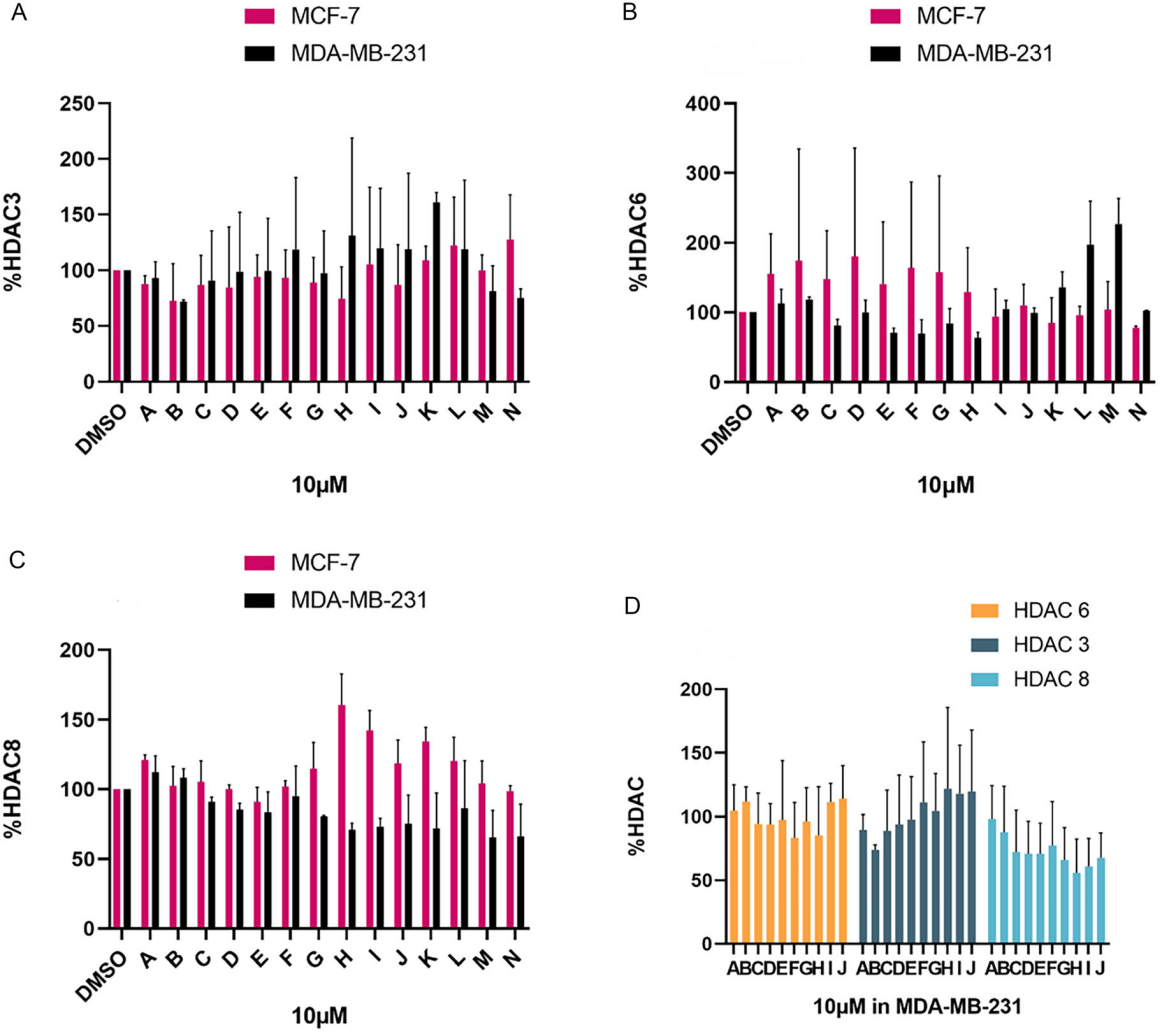
A) Compounds **A–N** were screened at 10 μM with HDAC3 abundance determined by quantitative western blotting in MCF‐7 and MDA‐MB‐231 cells; B) Compounds **A–N** were screened at 10 μM with HDAC6 abundance determined by quantitative western blotting in MCF‐7 and MDA‐MB‐231 cells; C) Compounds **A–N** were screened at 10 μM with HDAC8 abundance determined by quantitative western blotting in MCF‐7 and MDA‐MB‐231 cells; and D) Compounds **A–J** were screened at 10 μM with HDAC3/6/8 abundance determined by quantitative western blotting in MDA‐MB‐231 cells. Error bars represent the standard deviation (SD) of two independent biological replicates.

After initial screening, we further evaluated HDAC3/6/8 degradation activity in MCF‐7 and MDA‐MB‐231 cells at a lower concentration (10 μM) over 24 h, as shown in **Figure** **3** (western blots available in Figure S2 and S3). Notably, HDAC3 and HDAC6 degradation were markedly weaker than HDAC8. Notably, HDAC8 degradation was significantly stronger in MDA‐MB‐231 than in MCF‐7 cells after 24 h treatment at 10 μM. Meanwhile, linker length and composition strongly influenced degradation profiles, with carboxylic acid‐based linkers showing enhanced HDAC8 degradation in MDA‐MB‐231 cells. Among these compounds, **H** and **I** were the most potent HDAC8 degraders in the series, with significant activity at 10 μM (Figure 3D–**H**, **I**).

**Figure 4:**
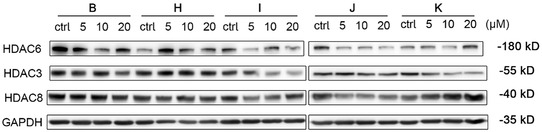
Compounds **B, H, I, J,** and **K** were screened at 5, 10, and 20 μM with HDAC6, 3, and 8 abundance determined by quantitative western blotting in MDA‐MB‐231 cells.

The most potent candidates (**B**, **H**, **I**, **J**, **K**) from each subseries were selected to evaluate their effects on HDAC3/6/8 degradation in MDA‐MB‐231 cells at 5, 10, and 20 μM (**Figure** **4**). Among these compounds, HDAC6 degradation was significantly greater than that of HDAC3 and HDAC8. Notably, compound **I** achieved ≈62% HDAC6 degradation at 5 μM, whereas compound **B** exhibited dose‐dependent effects–inducing ≈65% degradation at 10 μM but only ≈32% at the lower 5 μM concentration. Notably, compound **K** exhibited the obvious “hook effect” at the highest tested concentration (20 μM). Collectively, the data demonstrate that selected compounds exhibit HDAC degradation activity, with particularly potent effects against HDAC6.

### 2.3 Recognition of PROTAC by Polyamine Transporter

**Figure 5:**
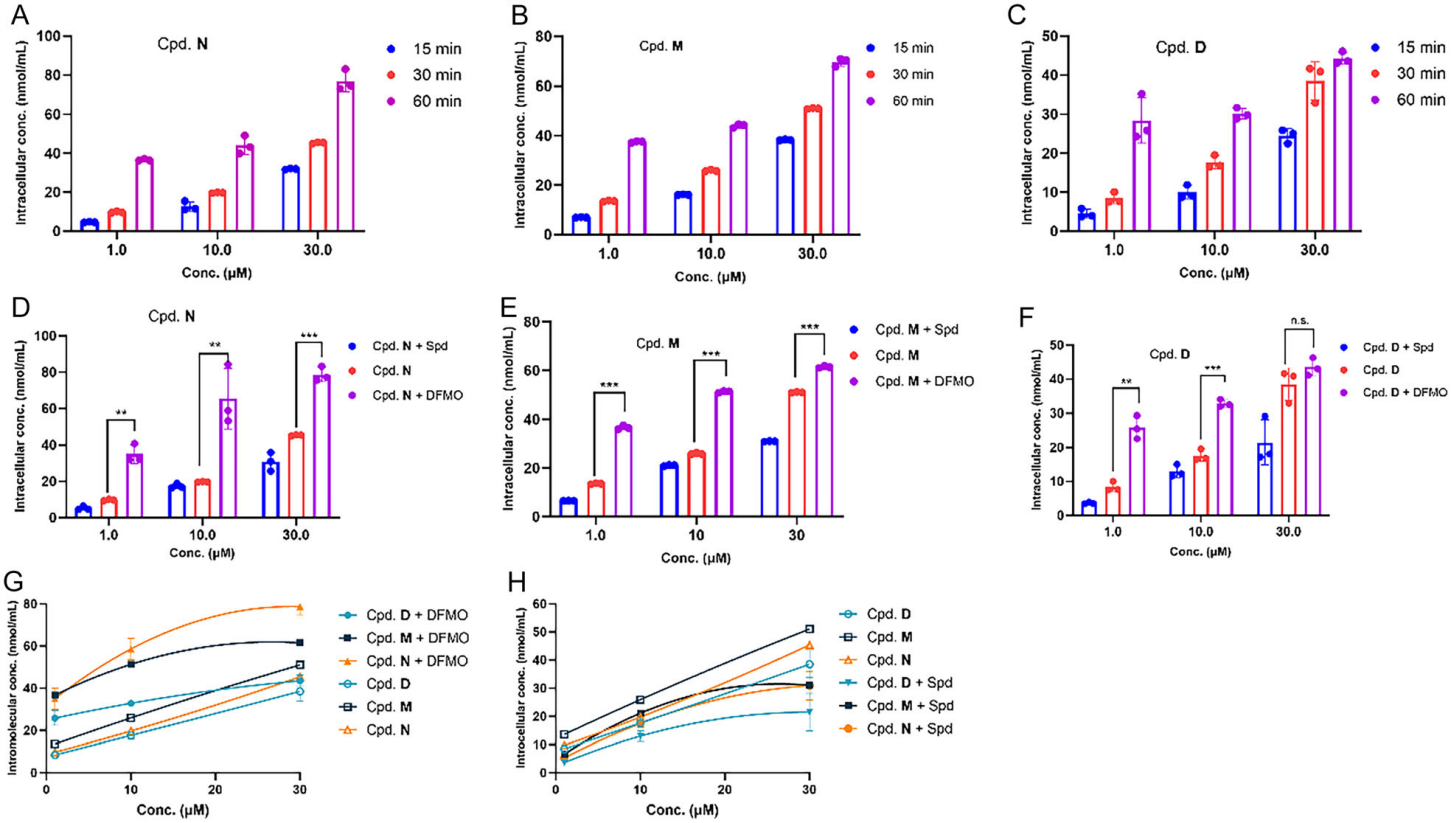
The intracellular concentrations of compounds **N, M,** and **D** were detected in MM1S cells by LC‐MS/MS. A) The intracellular concentrations of compound **N** was detected by LC‐MS/MS after treatment at 15, 30, and 60 min in MM1S cells; B) The intracellular concentrations of compound **M** was detected by LC‐MS/MS after treatment at 15, 30, and 60 min in MM1S cells; C) The intracellular concentrations of compound **D** was detected by LC‐MS/MS after treatment at 15, 30, and 60 min in MM1S cells; D) The intracellular concentrations of compound **N** was detected by LC‐MS/MS after co‐incubation with DFMO and Spd at 30 min in MM1S cells; E) The intracellular concentrations of compound **M** was detected by LC‐MS/MS after co‐incubation with DFMO and Spd at 30 min in MM1S cells; and F) The intracellular concentrations of compound **D** was detected by LC‐MS/MS after co‐incubation with DFMO and Spd at 30 min in MM1S cells. G,H) The intramolecular concentration comparison of three compounds (**N**, **M** and **D**) while the treatment of DFMO or Spd. Statistical analysis was performed using GraphPad Prism 8 (*n* = 3, $\bar{x}$ ± s).

To validate cellular uptake via the PTS, compounds **M, N,** and **D** were selected for LC‐MS/MS analysis. As shown in **Figure** **5**A–C, compounds **M**, **N**, and **D** exhibited dose‐ and time‐dependent increases in intracellular concentration. The maximum intracellular concentrations reached 70.8, 83.1, and 46.1 nmol/mL for compounds **M**, **N**, and **D**, respectively, after 60 min of treatment.

Tumor cells typically exhibit higher polyamine requirements for proliferation compared to normal cells. Difluoromethylornithine (DFMO), a polyamine biosynthesis inhibitor, enhances polyamine conjugate uptake via the polyamine transport (PAT). Spermidine (Spd), a natural substrate of the PAT, competitively inhibits drug uptake, significantly reducing intracellular drug accumulation. To determine whether PROTACs engage with PAT, we performed competitive uptake assays using either DFMO or Spd as reference PAT ligands.

As shown in Figure 5D–F, DFMO increased while Spd decreased intracellular concentrations of compounds **M**, **N**, and **D**, supporting their uptake via PTS. Furthermore, the combination of compound **N** with DFMO showed the greatest enhancement of PAT activity, increasing it by 73.4% compared to compound **N** alone (Figure 5D). Thus, we concluded that among these three PROTACs, compound **N** demonstrated the most efficient PTS‐mediated cellular uptake, likely due to optimal recognition of its Spd linker by the PAT system. Collectively, these findings demonstrate that polyamine linkers enhance PROTAC cellular uptake, providing valuable guidance for designing PROTACs with improved drug‐like properties.

## 3 Conclusion

In this study, we synthesized a series of HDAC‐PROTACs featuring diverse scaffolds, several of which exhibited HDAC degradation activity. Notably, compound **N**, which incorporates a polyamine linker, showed enhanced intracellular concentration via the PAT pathway. These findings provide valuable insights for designing HDAC‐PROTACs with improved membrane permeability and selectivity.

## 4 Experimental Section

4.1

4.1.1

##### Chemistry

General Methods. All reagents and solvents used were obtained from commercial suppliers (Bidepharmatech (Shanghai, China), Energy Chemical (The Woodlands, TX, USA), Alfa (Ronkonkoma, NY, USA), etc.) without further purification, except for special cases. Reactions were monitored via TLC. Thin‐layer chromatography was carried out using TLC silica gel 60 F254 plates. Flash column chromatography was performed with 200–300 mesh silica gel. The NMR spectrum was recorded on a Bruker‐400 NMR spectrometer, with TMS as an internal standard and chemical shifts reported in ppm (*δ*). Coupling constants (*J*) were reported in Hz. Spin multiplicities were described as s (singlet), br (broad singlet), d (doublet), t (triplet), q (quartet), and m (multiplet). An Agilent high‐performance liquid chromatography (HPLC) system was used for preparation. Additional figures for ^1^H NMR, ^13^C NMR, and LCMS of compounds used for biological testing were included in the Supporting Information.

##### Synthesis of Intermediate

((4‐((8‐(hydroxyamino)‐1,8‐dioxooctyl)amino)phenyl)oxy)acetic acid (**5**).

Synthesis of Intermediate **5** as Described in Scheme 1. To a solution of **1** (86.2 g, 458 mmol) and 4‐aminophenol (50.0 g, 458 mmol) in dry DCM (500 mL) at 0 °C, *N*,*N*‐diisopropylethyl‐amine (DIPEA) (159 mL, 913 mmol) and 2‐(7‐Azabenzotriazol‐1‐yl)‐*N*,*N*,*N*’,*N*’‐tetramethyluronium hexafluorophosphate (HATU) (174 g, 458 mmol) were added the reaction mixture was stirred at 15 °C overnight. The reaction mixture was diluted in DCM (600 mL × 3). The organic layer was dried over anhydrous sodium sulfate, filtered, and concentrated in vacuo to give the corresponding crude, which was stirred in ethyl acetate at 0 °C, then filtered to afford 2 as a white solid. To a solution of 2 in dry MeOH (600 mL), 50% NH_2_OH (321 g, 9.73 mol) was added. The reaction mixture was stirred at 60 °C for 12 h, then concentrated in vacuo to give the corresponding crude, which was purified by reversed‐phase HPLC (formic acid conditions) to afford **3** (25.0 g, 89.2 mmol, 36.7% yield) as a white solid.

To a solution of **3** (13.7 g, 48.9 mmol) and *tert*‐Butyl bromoacetate (9.53 g, 48.9 mmol) in dry dimethylformamide (DMF) (100 mL) and K_2_CO_3_ (10.8 g, 78.2 mmol) was added. The mixture was stirred at 20 °C for 12 h. The reaction was quenched by water (500 mL) and extracted with ethyl acetate (200 mL × 3). The combined organic layers were washed with brine (200 mL), dried over anhydrous sodium sulfate, and concentrated in vacuo. The residue was purified by silica gel column chromatography (petroleum ether/ethyl acetate = 2/1–0/1) to afford **4** (13.0 g, 33.0 mmol, 67.5% yield) as a red oil. TFA (11.7 mL) was added to a stirring solution of **4** (10.8 g, 27.4 mmol) in DCM (58 mL), and the mixture was stirred at 15 °C for 12 h. Then the mixture was concentrated in vacuo to give the corresponding crude, which was stirred in methyl *tert*‐butyl ether (500 mL × 3) and filtered to afford **5** (8.50 g, 25.1 mmol, 91.6% yield) as a white solid. ^1^H NMR (400 MHz, DMSO‐*d*_*6*_) *δ* 9.58 (s, 1H), 7.35 (d, *J* = 8.8 Hz, 2H), 6.67 (d, *J* = 8.8 Hz, 2H), 4.33 (s, 2H), 2.22 (t, *J* = 7.4 Hz, 2H), 1.98 (t, *J* = 7.0 Hz, 2H), 1.61–1.41 (m, 4H), 1.26 (s, 4H). ^13^C NMR (400 MHz DMSO‐*d*_*6*_) *δ* 170.94, 170.84, 170.41, 153.53, 131.51, 121.30 (2C), 115.43 (2C), 72.33, 36.66, 32.32, 28.87, 28.73, 25.59, 25.21. LCMS *m/z* : [M+H]^+^ calcd for C_16_H_23_N_2_O_6_, 339.15; found, 339.3.

##### General Procedures for the Synthesis of Compounds A, B, C, K, M, and N. Showing the Synthesis of Compound A as an Example

To a solution of **6a** (1.23 g, 4.45 mmol, 1.00 eq.) and *N*, *N*‐diisopropylethylamine (DIPEA) (2.30 g, 17.8 mmol, 3.10 mL, 4.00 eq.) in DMSO (15 mL) at 25 °C, then amine **7a** (1.00 g, 4.89 mmol, 1.10 eq.) was added. The reaction mixture was stirred at 90 °C for 6 h. The mixture was diluted with 25 mL of water, then extracted with ethyl acetate (100 mL × 2). The combined organic layer was washed with brine (100 mL × 2), dried over anhydrous sodium sulfate, and concentrated in vacuo to give the corresponding crude, which was chromatographically purified (petroleum ether/ethyl acetate = 50/1–1/1) to afford **8a** (435 mg, 0.945 mmol, 21.2% yield) as a yellow solid.

TFA (4 mL) was added to a stirring solution of **8a** (435 mg, 0.945 mmol) in DCM (8 mL), and the mixture was stirred at 25 °C for 4 h. The reaction mixture was concentrated in vacuo, suspended in ether (20 mL), and stirred at 25 °C for 12 h. The reaction mixture was filtered through a filter paper to afford **9a** (276 mg, 0.766 mmol, 81.1% yield) as a yellow solid.

To a solution of Compound **5** (670 mg, 1.98 mmol, 1.00 eq.) in dry DMF (8 mL), DIPEA (595 mg, 4.60 mmol, 802 μL, 6.00 eq.) and HATU (350 mg, 0.920 mmol, 1.20 eq.) were added. The reaction mixture was stirred for 30 min, after **9a** (276 mg, 0.766 mmol, 1.00 eq) was added. The reaction solution was stirred at 25 °C for 12 h. After that, 30 mL of water was added to quench and extracted with ethyl acetate (150 mL × 3); the organic layers were combined and washed with brine (125 mL × 2), dried over anhydrous sodium sulfate, filtered, and concentrated in vacuo to give the corresponding crude, which was chromatographically purified (methanol/ethyl acetate = 1/50–1/30) to afford **A** (185 mg, 0.272 mmol, 35.5% yield) as a yellow solid.

8‐((4‐((1‐((2‐(2,6‐dioxohexahydropyridin‐3‐yl)‐1,3‐dioxo‐2,3‐dihydro‐1*H*‐isoindol‐4‐yl)amino)‐7‐oxo‐6‐aza‐3‐oxaoct‐8‐yl)oxy)phenyl)amino)‐8‐oxooctan‐1‐hydroxamic acid (**A**).

^1^H NMR(400 MHz, MeOD‐*d*_*4*_) *δ* 11.24 (brs, 1H), 10.82 (brs, 1H), 9.52 (brs, 1H), 9.12 (brs, 1H), 8.67 (brs, 1H), 7.73 (brs, 1H), 7.53 (t, *J* = 8.0 Hz, 1H), 7.32 (d, *J* = 8.4 Hz, 2H), 7.06 (dd, *J* = 10.8, 8.2 Hz, 2H), 6.74 (d, *J* = 8.0 Hz, 2H), 5.07 (dd, *J* = 12.0, 4.0 Hz, 1H), 4.34–4.29 (m, 2H), 3.71–3.47 (m, 8H), 3.01–2.68 (m, 3H), 2.31 (t, *J* = 8.0 Hz, 2H), 2.07 (t, *J* = 8.0 Hz, 2H), 2.03 (s, 1H), 1.66–1.59 (m, 4H), 1.34–1.31 (m, 4H). ^13^C NMR (101 MHz, MeOD‐*d*_*4*_) *δ* 174.69, 174.21, 173.95, 171.77, 171.16, 170.67, 169.30, 155.27, 148.19, 137.22, 133.79, 131.70, 123.33, 118.31, 116.19, 112.02, 111.25, 76.27, 70.35, 70.17, 61.54, 50.32, 43.19, 40.03, 37.64, 33.40, 32.19, 29.82, 29.74, 26.74, 26.18, 23.79, 14.45. LCMS *m/z*: [M+H]^+^ calcd. for C_33_H_41_N_6_O_10_, 681.2; found, 681.5.

8‐((4‐((1‐((2‐(2,6‐dioxohexahydropyridin‐3‐yl)‐1,3‐dioxo‐2,3‐dihydro‐1*H*‐isoindol‐4‐yl)amino)‐10‐oxo‐9‐aza‐3,6‐dioxaundec‐11‐yl)oxy)phenyl)amino)‐8‐oxooctan‐1‐hydroxamic acid (**B**).

^1^H NMR (400 MHz, DMSO‐*d*_*6*_) *δ* 11.30 (brs, 1H), 11.09 (s, 1H), 9.56 (s, 1H), 9.11 (brs, 1H), 8.37 (brs, 1H), 7.60–7.56 (m, 1H), 7.34 (d, *J* = 8.8 Hz, 2H), 7.14 (d, *J* = 8.6 Hz, 1H), 7.04 (d, *J* = 7.0 Hz, 1H), 6.66 (d, *J* = 8.8 Hz, 2H), 6.62 (m, 1H), 5.06 (dd, *J* = 12.8, 5.4 Hz, 1H), 4.21 (s, 2H), 3.63–3.52 (m, 10H), 3.28–3.24 (m, 2H), 2.91–2.83 (m, 2H), 2.33 (m, 1H), 2.21 (t, *J* = 7.4 Hz, 2H), 2.08–1.96 (m, 3H), 1.51 (br dd, *J* = 7.2, 14.8 Hz, 4H), 1.25 (s, 4H). ^13^C NMR (400 MHz DMSO‐*d*_*6*_) *δ* 173.30, 170.91 170.58,169.43, 168.39, 167.79 (2C), 153.54, 146.89, 136.71, 132.58, 131.53, 121.29, 121.19, 117.92, 115.43, 111.15, 109.74, 75.09, 70.15, 70.08 (2C), 69.37, 69.35, 49.03, 42.16, 38.70, 38.58, 32.37, 31.46, 28.86, 28.72, 25.57, 25.16, 22.60. LCMS *m/z*: [M+H]^+^ calcd. for C_35_H_45_N_6_O_11_, 725.3; found, 725.5.

8‐((4‐((1‐((2‐(2,6‐dioxohexahydropyridin‐3‐yl)‐1,3‐dioxo‐2,3‐dihydro‐1*H*‐isoindol‐4‐yl)amino)‐13‐oxo‐12‐aza‐3,6,9‐trioxatetradec‐14‐yl)oxy)phenyl)amino)‐8‐oxooctan‐1‐hydroxamic acid (**C**).

^1^H NMR (400 MHz, MeOD‐*d*_*4*_) *δ* 7.53 (dd, *J* = 8.8, 7.2 Hz, 1H), 7.30 (d, *J* = 9.2 Hz, 2H), 7.05 (dd, *J* = 11.2, 8.8 Hz, 2H), 6.72 (d, *J* = 8.8 Hz, 2H), 4.56 (s, 1H), 4.30 (s, 2H), 3.70 (t, *J* = 8.8 Hz, 2H), 3.64–3.54 (m, 10H), 3.48 (t, *J* = 5.6 Hz, 2H), 3.41 (t, *J* = 5.6 Hz, 2H), 2.86–2.68 (m, 4H), 2.30 (t, *J* = 7.6 Hz, 2H), 2.11–2.07 (m, 2H), 1.71–1.57 (m, 4H), 1.36–1.34 (m, 4H). ^13^C NMR (101 MHz, MeOD‐*d*_*4*_) *δ* 174.68, 174.20, 171.62, 170.65, 169.30, 155.28, 148.20, 137.21, 133.86, 131.73, 123.34 (2C), 118.28, 116.20 (2C), 112.03, 111.28, 76.18, 71.64 (3C), 71.62 (2C), 71.35, 70.61, 70.26, 50.20, 43.25, 40.05, 37.66, 33.45, 32.21, 29.85, 29.77, 26.77, 26.23, 23.81. LCMS m/z: [M+H]^+^ calcd. for C_37_H_49_N_6_O_12_, 769.3;found, 769.5.

8‐((4‐((1‐((2‐(2,6‐dioxohexahydropyridin‐3‐yl)‐1,3‐dioxo‐2,3‐dihydro‐1*H*‐isoindol‐5‐yl)amino)‐7‐oxo‐6‐aza‐3‐oxaoct‐8‐yl)oxy)phenyl)amino)‐8‐oxooctan‐1‐hydroxamic acid (**K**).

^1^H NMR (400 MHz, MeOD‐ *d*_*4*_) *δ* 7.54 (d, *J* = 8.4 Hz, 1H), 7.29 (d, *J* = 8.8 Hz, 2H), 7.02 (d, *J* = 1.9 Hz, 1H), 6.86 (dd, *J* = 8.4, 1.9 Hz, 1H), 6.71 (d, *J* = 8.8 Hz, 2H), 5.03 (dd, *J* = 12.5, 5.4 Hz, 1H), 4.32 (s, 2H), 3.69 (t, *J* = 5.1 Hz, 2H), 3.58 (t, *J* = 5.1 Hz, 2H), 3.45 (t, *J* = 5.0 Hz, 2H), 3.39 (t, *J* = 5.1 Hz, 2H), 2.88–2.63 (m, 3H), 2.28 (t, *J* = 7.4 Hz, 2H), 2.13–2.05 (m, 3H), 1.62 (d, *J* = 7.1 Hz, 4H), 1.33 (s, 4H). ^13^C NMR (101 MHz, MeOD‐ *d*_*4*_) *δ* 173.32, 172.82, 171.61, 170.41, 169.67, 168.20, 167.90, 154.69, 153.91, 134.44, 130.30, 124.84, 121.97, 117.03, 115.35, 114.81, 105.87, 74.97, 68.97, 68.70, 60.15, 42.66, 38.68, 36.27, 32.07, 30.82, 28.48, 28.40, 25.37, 24.88, 22.45, 19.48, 13.08.LCMS m/z: [M+H]^+^ calcd. for C_33_H_41_N_6_O_10_, 681.3; found, 681.4.

8‐((4‐((8‐((2‐(2,6‐dioxohexahydropyridin‐3‐yl)‐1,3‐dioxo‐2,3‐dihydro‐1*H*‐isoindol‐4‐yl)amino)‐2‐oxo‐3,6‐diazaoct‐1‐yl)oxy)phenyl)amino)‐8‐oxooctan‐1‐hydroxamic acid (**M**).

^1^H NMR (400 MHz, MeOD*‐d*_*4*_) *δ* 7.57–7.54 (m, 1H), 7.30 (d, *J* = 8.5 Hz, 2H), 7.07 (t, *J* = 7.4 Hz, 2H), 6.71 (d, *J* = 8.7 Hz, 2H), 5.05 (dd, *J* = 12.6, 5.6 Hz, 1H), 4.31 (s, 2H), 3.52–3.43 (m, 4H), 3.01–2.68 (m, 6H), 2.30 (t, *J* = 7.4 Hz, 2H), 2.11–2.05 (m, 2H), 1.92 (s, 2H), 1.70–1.57 (m, 4H), 1.40–1.31 (m, 4H). ^13^C NMR (101 MHz, MeOD*‐d*_*4*_) *δ* 173.34, 172.94, 170.73, 169.72, 153.94, 135.01, 133.15, 132.52, 130.25, 128.69, 126.48, 122.07, 120.09, 114.82, 52.28, 38.39, 36.25, 35.32, 31.98, 31.65, 30.96, 29.34, 29.19, 28.91, 28.41, 28.34, 26.71, 25.37, 24.81, 22.69, 22.61, 22.33, 13.05. LCMS m/z : [M+H]^+^ calcd. for C_33_H_42_N_7_O_9_, 680.3; found, 679.7.

8‐((4‐((11‐((2‐(2,6‐dioxohexahydropyridin‐3‐yl)‐1,3‐dioxo‐2,3‐dihydro‐1*H*‐isoindol‐4‐yl)amino)‐2‐oxo‐3,7‐diazaundec‐1‐yl)oxy)phenyl)amino)‐8‐oxooctan‐1‐hydroxamic acid (**N**).

^1^H NMR (400 MHz, MeOD*‐d*_*4*_) *δ* 7.51 (dd, *J* = 8.5, 7.2 Hz, 1H), 7.30 (d, *J* = 8.9 Hz, 2H), 7.05–7.02 (m, 2H), 6.72 (d, *J* = 8.9 Hz, 2H), 5.05 (dd, *J* = 12.5, 5.4 Hz, 1H), 4.30 (s, 2H), 3.71 (t, *J* = 5.2 Hz, 2H), 3.64 (d, *J* = 2.1 Hz, 4H), 3.58 (t, *J* = 5.5 Hz, 2H), 3.44 (dt, *J* = 16.4, 5.4 Hz, 4H), 2.89–2.64 (m, 3H), 2.30 (t, *J* = 7.4 Hz, 2H), 2.13–2..06 (m, 3H), 1.68–1.55 (m, 5H), 1.39–1.23 (m, 5H). ^13^C NMR (101 MHz, MeOD*‐d*_*4*_) *δ* 174.66, 174.51, 174.24, 172.08, 171.06, 170.82, 155.32, 150.95, 140.76, 136.33, 134.58, 133.90, 131.65, 130.03, 129.47, 127.84, 123.39, 121.76, 121.38, 116.18, 76.24, 53.66, 40.40, 39.93, 37.64, 37.10, 33.40, 32.34, 29.94, 29.83, 29.72, 27.50, 26.76, 26.37, 26.21, 24.09. LCMS m/z: [M+H]^+^ calcd. for C_36_H_48_N_7_O_9_, 722.3; found, 721.7.

##### General Procedures for the Synthesis of Compounds D, E, F, and L

To a solution of **6a** (750 mg, 2.72 mmol, 1.00 eq.) and *N*, *N*‐diisopropylethylamine (DIPEA) (1.05 g, 10.9 mmol, 1.42 mL, 3.00 eq.) in N‐Methyl‐2‐pyrrolidone (NMP) (15 mL) at 25 °C, then amine **7e** (603 mg, 2.99 mmol, 1.10 eq.) was added. The reaction mixture was stirred at 90 °C for 12 h. The mixture was diluted with 40 mL of water, then extracted with ethyl acetate (150 mL × 2). The combined organic layer was washed with brine (100 mL × 3), dried over anhydrous sodium sulfate, and concentrated in vacuo. The residue was purified by silica gel column chromatography (petroleum ether/ethyl acetate = 5/1–1/1) to afford **8e** (1.06 g, 2.31 mmol, 85.1% yield) as a green oil.

TFA (2 mL) was added to a stirring solution of **8e** (1.00 g, 2.18 mmol) in DCM (5 mL), and the mixture was stirred at 25 °C for 4 h. The reaction mixture was concentrated in vacuo, suspended in ether (15 mL), and stirred at 25 °C for 12 h. The reaction mixture was filtered through a filter paper to afford **9e** (752 mg, 2.10 mmol, 96.3% yield) as a yellow solid.

To a solution of Compound **5** (670 mg, 1.98 mmol, 1.00 eq.) in dry DMSO (10 mL), *N*‐hydroxy‐7‐azabenzotriazole (HOAt) (404 mg, 2.97 mmol, 1.50 eq.), *N*‐methyl morpholine (NMM) (604 mg, 5.94 mmol, 657 μL 3.00 eq.), and 1‐(3‐dimethylaminopropyl)‐3‐ethylcarbodiimide hydrochloride (EDCI) (569 mg, 2.97 mmol, 1.50 eq.) were added at 25 °C. The reaction mixture was stirred for 30 min, after **9e** (710 mg, 1.98 mmol, 1.00 eq.) was added. The reaction solution was stirred at 25 °C for 12 h. After that, 40 mL of water was added to quench and extracted with ethyl acetate (250 mL × 2); the organic layers were combined and washed with brine (100 mL × 2), dried over anhydrous sodium sulfate, filtered, and concentrated in vacuo to give the corresponding crude, which was chromatographically purified (methanol/ethyl acetate = 1/50–1/10) to afford **E** (204 mg, 0.30 mmol, 15.2% yield) as a yellow solid.

8‐((4‐((2‐((5‐((2‐(2,6‐dioxohexahydropyridin‐3‐yl)‐1,3‐dioxo‐2,3‐dihydro‐1*H*‐isoindol‐4‐yl)amino)pentyl)amino)‐2‐oxoethyl)oxy)phenyl)amino)‐8‐oxooctan‐1‐hydroxamic acid (**E**).

^1^H NMR (400 MHz, MeOD‐*d*_*4*_) *δ* 10.84 (brs, 1H), 9.55 (brs, 1H), 8.77 (d, *J* = 3.8 Hz, 1H), 8.46 (d, *J* = 8.2 Hz, 1H), 7.75 (s, 1H), 7.65 (s, 1H), 7.55 (t, *J* = 7.7 Hz, 1H), 7.35 (d, *J* = 8.6 Hz, 2H), 7.06–7.02 (m, 2H), 6.76 (d, *J* = 8.7 Hz, 2H), 4.34–4.31 (m, 3H), 3.34 (d, *J* = 9.1 Hz, 4H), 2.93–2.70 (m, 3H), 2.33 (t, *J* = 7.3 Hz, 2H), 2.13 (t, *J* = 7.1 Hz, 3H), 1.79–1.63 (m, 9H), 1.49 (q, *J* = 7.4 Hz, 4H), 1.28 (t, *J* = 7.1 Hz, 1H). ^13^C NMR (101 MHz, MeOD‐*d*_*4*_) *δ* 174.65, 174.15, 174.06, 171.72, 170.84, 170.74, 169.30, 155.28, 148.19, 137.24, 133.85, 132.33, 131.70, 129.85, 123.30, 118.00, 116.18, 111.75, 110.94, 76.28, 66.64, 43.24, 39.83, 37.64, 33.42, 32.20, 31.69, 29.86, 26.75, 26.23, 25.12, 23.79, 20.24, 14.46.LCMS *m/z*: [M+H]^+^ calcd. for C_34_H_43_N_6_O_9_, 679.3; found, 679.7.

8‐(4‐(2‐((4‐((2‐(2,6‐dioxopiperidin‐3‐yl)‐1,3‐dioxoisoindolin‐4‐yl)a‐mino)butyl)amino)‐2‐oxoethoxy)phenyl)amino)‐8‐oxooctan‐1‐hydroxamic acid (**D**).

^1^H NMR (400 MHz, MeOD‐*d*_*4*_) *δ* 7.55 (dd, *J* = 8.5, 7.2 Hz, 1H), 7.32 (d, *J* = 8.9 Hz, 2H), 7.07–7.03 (m, 2H), 6.73 (d, *J* = 8.9 Hz, 2H), 5.06 (dd, *J* = 12.5, 5.4 Hz, 1H), 4.33 (s, 2H), 3.38–3.34 (m, 4H), 2.91–2.82 (m, 1H), 2.76–2.67 (m, 2H), 2.31 (t, *J* = 7.4 Hz, 2H), 2.11 (t, *J* = 7.2 Hz, 3H), 1.69–1.61 (m, 8H), 1.37–1.35 (m, 4H). ^13^C NMR (101 MHz, MeOD‐*d*_*4*_) *δ* 173.24, 172.81, 170.24, 169.53, 169.35, 167.94, 153.93, 146.82, 135.86, 132.53, 130.32, 121.99, 116.66, 114.80, 110.42, 109.69, 74.92, 48.81, 41.67, 38.28, 36.24, 32.04, 30.80, 28.43, 28.36, 26.28, 26.19, 25.34, 24.82, 22.41. LCMS *m/z*: [M+H]^+^ calcd. for C_33_H_41_N_6_O_9_, 665.3; found, 665.3.

8‐((4‐((2‐((6‐((2‐(2,6‐dioxohexahydropyridin‐3‐yl)‐1,3‐dioxo‐2,3‐dihydro‐1*H*‐isoindol‐4‐yl)amino)hexyl)amino)‐2‐oxoethyl)oxy)phenyl)amino)‐8‐oxooctan‐1‐hydroxamic acid (**F**).

^1^H NMR (400 MHz, MeOD‐*d*_*4*_) *δ* 7.72 (dd, *J* = 5.7, 3.3 Hz, 2H), 7.61 (dd, *J* = 5.7, 3.3 Hz, 2H), 7.53(t, *J* = 7.8 Hz, 1H), 7.30 (d, *J* = 8.9 Hz, 2H), 7.03–7.01 (m, 2H), 6.71 (d, *J* = 8.9 Hz, 2H),, 4.31–4.29 (m, 5H), 3.25 (t, *J* = 6.9 Hz, 2H), 2.89–2.65 (m, 3H), 2.30 (t, *J* = 7.5 Hz, 2H), 2.10 ((t, *J* = 7.4 Hz, 3H), 1.76–1.70 (m, 4H), 1.48–1.43 (m, 6H), 0.98 (t, *J* = 7.4 Hz, 6H). ^13^C NMR (101 MHz, MeOD‐*d*_*4*_) *δ* 174.68, 174.19, 171.70, 170.78, 169.35, 169.32, 155.30, 148.28, 137.24, 133.90, 133.56, 132.35, 131.72, 129.86, 123.31, 118.02, 116.18, 111.72, 110.95, 76.27, 66.66, 43.31, 40.01, 37.65, 33.43, 32.20, 31.71, 30.20, 29.86, 27.63, 26.77, 26.25, 23.80, 20.25, 14.04. LCMS *m/z*: [M+H]^+^ calcd. for C_35_H_45_N_6_O_9_, 693.3; found, 693.4.

8‐((4‐((2‐((5‐((2‐(2,6‐dioxohexahydropyridin‐3‐yl)‐1,3‐dioxo‐2,3‐dihydro‐1*H*‐isoindol‐5‐yl)amino)pentyl)amino)‐2‐oxoethyl)oxy)phenyl)amino)‐8‐oxooctan‐1‐hydroxamic acid ethane hydrate (**L**).

^1^H NMR (400 MHz, MeOD‐*d*_4_) *δ* 7.54 (d, *J* = 8.3 Hz, 1H), 7.32 (d, *J* = 8.4 Hz, 2H), 6.96 (s, 1H), 6.81 (d, *J* = 7.8 Hz, 1H), 6.73 (d, *J* = 8.5 Hz, 2H), 5.05 (dd, *J* = 12.3, 5.1 Hz, 1H), 4.32 (s, 2H), 3.29 (t, *J* = 6.2 Hz, 2H), 3.19 (t, *J* = 6.3 Hz, 2H), 2.90–2.65 (m, 3H), 2.31 (t, *J* = 7.2 Hz, 2H), 2.13–2.10 (m, 3H), 1.67–1.59 (m, 8H), 1.48 (d, *J* = 6.5 Hz, 2H), 1.36 (s, 4H). ^13^C NMR (101 MHz, MeOD‐*d*_*4*_) *δ* 173.33, 172.81, 171.61, 170.43, 169.44, 168.27, 167.93, 154.75, 153.91, 134.52, 130.30, 124.86, 121.98, 116.55, 114.81, 105.40, 74.93, 60.16, 48.91, 42.63, 38.52, 36.27, 32.08, 30.83, 28.55, 28.49, 28.40, 28.00, 25.38, 24.88, 23.96, 22.46, 19.49, 13.08. LCMS *m/z*: [M+H]^+^ calcd. for C_34_H_43_N_6_O_9_, 679.3; found, 678.3.

##### General Procedures for the Synthesis of Compounds G, H, I, and J

To a solution of Compound **10** (500 mg, 1.93 mmol, 1.00 eq.) in dry DMSO (6 mL) at room temperature, *N*‐hydroxy‐7‐azabenzotriazole (HOAT) (395 mg, 2.90 mmol, 1.50 eq.), *N*‐methyl morpholine (NMM) (586 mg, 5.79 mmol, 637 μL 3.00 eq.), and 1‐(3‐dimethylaminopropyl)‐3‐ethylcarbodiimide hydrochloride (EDCI) (556 mg, 2.90 mmol, 1.50 eq.) were added. The reaction mixture was stirred for 30 min, after **11b** (392 mg, 1.93 mmol, 1.00 eq.) was added. The reaction solution was stirred at room temperature overnight. After that, 15 mL of water was added to quench and extracted with ethyl acetate (150 mL × 3), the organic layers were combined and washed with brine (100 mL × 2), dried over anhydrous sodium sulfate, filtered, and concentrated in vacuo to give the corresponding crude, which was chromatographically purified (petroleum ether/ethyl acetate = 1/5–0/1) to afford **12b** (707 mg, 1.59 mmol, 82.4% yield) as a white solid.

TFA (5 mL) was added to a stirring solution of **12b** (550 mg, 1.24 mmol) in DCM (10 mL), and the mixture was stirred at 25 °C for 4 h. The reaction mixture was concentrated in vacuo, suspended in ether (20 mL), and stirred at 25 °C for 12 h. The reaction mixture was filtered through a filter paper to afford **13b** (401 mg, 0.904 mmol, 72.9% yield) as a light‐yellow solid.

To a solution of Compound **5** (294 mg, 0.870 mmol, 1.00 eq.) in dry DMF (6 mL), DIPEA (900 mg, 6.96 mmol, 1.21 mL, 8.00 eq.) and HATU (395 mg, 1.04 mmol, 1.20 eq.) were added. The reaction mixture was stirred for 30 min, after **13b** (300 mg, 0.870 mmol, 1.00 eq.) was added. The reaction solution was stirred at 25 °C overnight. After that, 20 mL of water was added to quench and extracted with ethyl acetate (175 mL × 3); the organic layers were combined and washed with brine (100 mL × 3), dried over anhydrous sodium sulfate, filtered, and concentrated in vacuo to give the corresponding crude, which was chromatographically purified (methanol/ethyl acetate = 1/30–1/20) to afford **H** (144 mg, 0.220 mmol, 25.3% yield) as a white solid.

8‐((4‐((2‐((4‐((2‐(2,6‐dioxohexahydropyridin‐3‐yl)‐1‐oxo‐2,3‐dihydro‐1*H*‐isoindol‐4‐yl)amino)‐4‐oxobutyl)amino)‐2‐oxoethyl)oxy)phenyl)amino)‐8‐oxooctan‐1‐hydroxamic acid (**H**).

^1^H NMR (400 MHz, MeOD‐*d*_*4*_) *δ* 8.73 (d, *J* = 4.3 Hz, 1H), 8.43 (d, *J* = 8.3 Hz, 1H), 7.74 (d, *J* = 8.1 Hz, 1H), 7.64 (d, *J* = 7.4 Hz, 1H), 7.51 (d, *J* = 8.2 Hz, 1H), 7.29 (d, *J* = 8.8 Hz, 2H), 6.71 (d, *J* = 8.8 Hz, 2H), 4.49 (s, 2H), 4.31–4.07 (m, 3H), 3.35 (t, *J* = 6.7 Hz, 2H), 2.94–2.85(m, 1H), 2.78–2.74 (m, 1H), 2.51–2.42 (m, 3H), 2.28 (t, *J* = 7.4 Hz, 2H), 2.20–2.08 (m, 3H), 1.97–1.87 (m, 2H), 1.75–1.40 (m, 7H), 1.24 (t, *J* = 7.1 Hz, 1H). ^13^C NMR (101 MHz, MeOD‐*d*_*4*_) *δ* 174.65, 174.22, 173.74, 172.06, 171.07, 171.00, 155.32, 152.15, 134.55, 133.90, 132.33, 131.65, 130.05, 129.93, 129.85, 137.55, 123.38, 122.14, 116.18, 76.33, 66.65, 39.36, 37.61, 34.43, 33.40, 32.35, 31.70, 29.83, 29.75, 26.74, 26.38, 24.09, 20.25. LCMS m/z: [M+H]^+^ calcd. for C_33_H_41_N_6_O_9_, 665.3; found, 665.3.

8‐((4‐((2‐((3‐((2‐(2,6‐dioxohexahydropyridin‐3‐yl)‐1‐oxo‐2,3‐dihydro‐1*H*‐isoindol‐4‐yl)amino)‐3‐oxopropyl)amino)‐2‐oxoethyl)oxy)phenyl)amino)‐8‐oxooctan‐1‐hydroxamic acid (**G**).

^1^H NMR: (400 MHz, MeOD‐*d*_*4*_) *δ* 7.77–7.72 (m, 1H), 7.68–7.65 (m, 1H), 7.53 (t, *J* = 7.7 Hz, 1H), 7.33–7.31 (m, 2H), 6.75–6.72 (m, 2H), 5.18 (dd, *J* = 5.2, 13.3 Hz, 1H), 4.54–4.48 (m, 2H), 4.33 (s, 2H), 3.65 (t, *J* = 6.2 Hz, 2H), 2.96–2.87 (m, 1H), 2.81–2.75 (m, 1H), 2.70 (t, *J* = 6.4 Hz, 2H), 2.50 (qt, *J* = 4.6, 13.3 Hz, 1H), 2.31 (t, *J* = 7.5 Hz, 2H), 2.22–2.16 (m, 1H), 2.07–2.03 (m, 2H), 1.67–1.54 (m, 4H), 1.36–1.31 (m, 4H) ***.*** ^13^C NMR: (101 MHz, MeOD‐*d*_*4*_) *δ* 173.22, 172.87, 170.82, 170.60, 170.60, 170.52, 169.71, 153.95, 135.23, 133.10, 132.56, 130.31, 128.63, 126.49, 122.04 (2C), 120.14, 114.81 (2C), 74.97, 52.21, 36.23, 35.42, 35.33, 35.20, 32.00, 30.95, 28.38, 28.34, 25.32, 24.72, 22.72. LCMS m/z: [M+H]^+^ calcd. for C_32_H_39_N_6_O_9_, 651.3; found, 651.2.

8‐((4‐((2‐((5‐((2‐(2,6‐dioxohexahydropyridin‐3‐yl)‐1‐oxo‐2,3‐dihydro‐1*H*‐isoindol‐4‐yl)amino)‐5‐oxopentyl)amino)‐2‐oxoethyl)oxy)phenyl)amino)‐8‐oxooctan‐1‐hydroxamic acid (**I**).

^1^H NMR (400 MHz, MeOD‐*d*_*4*_) *δ* 9.78 (brs, 1H), 9.47 (brs, 1H), 9.05 (brs, 1H), 8.63 (brs, 1H), 7.63 (d, *J* = 7.9 Hz, 1H), 7.55 (d, *J* = 7.5 Hz, 1H), 7.41 (t, *J* = 7.7 Hz, 1H), 7.20 (d, *J* = 8.8 Hz, 2H), 6.61 (d, *J* = 8.8 Hz, 2H), 4.51 (s, 1H), 4.39–4.38 (m, 2H), 4.24 (s, 2H), 2.84–2.64 (m, 2H), 2.41–2.36 (m, 3H), 2.20 (t, *J* = 7.4 Hz, 2H), 2.12–1.91 (m, 5H), 1.67–1.46 (m, 9H), 1.19 (s, 3H). ^13^C NMR (101 MHz, MeOD‐*d*_*4*_) *δ* 174.73, 174.32, 172.11, 171.11, 155.32, 136.40, 134.54, 133.91, 131.64, 130.87, 130.79, 130.08, 127.87, 123.45, 121.48, 116.21, 39.78, 37.64, 36.70, 33.37, 33.04, 32.35, 30.73, 30.57, 30.30, 29.80, 29.73, 28.10, 26.76, 26.20, 24.08, 24.00, 23.71, 14.44. LCMS m/z: [M+H]^+^ calcd. for C_34_H_43_N_6_O_9_, 679.3; found, 679.3.

8‐((4‐((2‐((6‐((2‐(2,6‐dioxohexahydropyridin‐3‐yl)‐1‐oxo‐2,3‐dihydro‐1*H*‐isoindol‐4‐yl)amino)‐6‐oxohexyl)amino)‐2‐oxoethyl)oxy)phenyl)amino)‐8‐oxooctan‐1‐hydroxamic acid (**J**).

^1^H NMR (400 MHz, MeOD‐*d*_*4*_) *δ* 7.71 (d, *J* = 7.9 Hz, 1H), 7.64 (d, *J* = 7.4 Hz, 1H), 7.50 (t, *J* = 7.7 Hz, 1H), 7.29 (d, *J* = 8.8 Hz, 2H), 6.71 (d, *J* = 8.8 Hz, 2H), 5.14 (dd, *J* = 13.3, 5.1 Hz, 1H), 4.47 (s, 2H), 4.29 (s, 2H), 3.26 (t, *J* = 6.9 Hz, 2H), 2.93–2.73 (m, 2H), 2.44 (t, *J* = 7.4 Hz, 3H), 2.30 (t, *J* = 7.4 Hz, 2H), 2.18–2.13 (m, 1H), 2.08 (t, *J* = 7.3 Hz, 2H), 1.77–1.70 (m, 2H), 1.67–1.55 (m, 6H), 1.47–1.41 (m, 2H), 1.39–1.28 (m, 4H). ^13^C NMR (101 MHz, MeOD‐*d*_*4*_ *) δ* 173.28, 173.09, 172.84, 171.61, 170.70, 169.68, 169.42, 153.94, 134.99, 133.20, 132.54, 130.27, 128.67, 126.46, 122.01, 120.03, 114.81, 74.87, 60.15, 52.27, 38.54, 36.26, 35.73, 32.03, 30.95, 28.58, 28.47, 28.36, 26.14, 25.38, 25.00, 24.85, 22.71, 19.47, 13.07. LCMS m/z: [M+H]^+^ calcd. for C_35_H_45_N_6_O_9_, 693.3; found, 692.8.

##### Cell Culture

Cell lines were purchased from American Type Culture Collection (ATCC) unless otherwise noted. HCT‐116, MDA‐MB‐231, and MCF‐7 cells were cultured in DMEM medium (Corning, 4.5 g L^−1^ glucose) supplemented with 10% FBS and 1% Penicillin/Streptomycin. MM1S were cultured in PRMI‐1640 medium supplemented with 10% FBS, 1% Penicillin/Streptomycin. All cell lines were grown at 37 °C in a humidified 5% CO_2_ atmosphere.

##### Western Blot and Protein Degradation Assay

When the cells reached 90% confluence, they were seeded in 12‐well plates (020012, In Vitro Scientific, Hangzhou, China), incubated until cells reached about 40% confluence, and treated with compounds at indicated concentrations for 24 h. Compounds were dissolved in DMSO for storage (10 mM). Following compound treatment, cells were lysed using 2.5 × loading buffer (6 × 10^5^ cells/100 μL Loading buffer; 5× loading buffer: 10% SDS, 5% *β*‐mercaptoethanol, 50% glycerin, 1% bromophenol blue, 6% 1 M Tris HCl) and transferred to a 1.5 mL Eppendorf tube, then heated at 95 °C for 20 min. Equal quantities of cell lysates were separated using a 10% SDS–PAGE gel and transferred to PVDF membranes (IPVH00010, Millipore; Merck KGaA). The PVDF membranes were blocked with 5% defatted milk in 1 × TBST for 1 h at room temperature and incubated with certain primary antibodies at 4 °C overnight. PVDF membranes were washed three times with 1 × TBST for 10 min each and then incubated with the appropriate HRP‐conjugated secondary antibody for 1 h at room temperature. After washing three more times with 1 × TBST, the PVDF membrane was stained with a hypersensitive ECL chemiluminescence reagent (Cat. FD8000, Fdbio science, Hangzhou, China) and then imaged with a chemiluminescence imaging system (GE Amersham ImageQuant 800, USA). Grayscale quantitative analysis was performed using ImageJ software. Blotting analysis was performed by GraphPad Prism 8. Mean ± SD and unpaired t tests were performed in GraphPad Prism 8.

##### Polyamine Transport Ability Assay

MM1S cells were harvested and plated with 2 × 10^5^ cells in 500 μL media per well in 24‐well plates. After overnight seeding, 500 μL media containing different dosing concentrations (1, 10, 30 μM) of the compounds were added to each well alone or combined with DFMO (100 μM) and Spd (500 μM). After treatment for different times (15, 30, 60 min), cells were moved to an Eppendorf tube and the supernatant was removed by centrifugation for 30 min at 12 000 r min^−1^ at 4°C. Then cells were washed twice with phosphate buffer saline to remove the incubation medium and subsequently lysed with 50 μL RIPA Lysis Buffer and shaken on an ice bath for 30 min. After adding 50 μL of MeOH, the supernatant was separated by centrifugation for 30 min at 12 000 r min^−1^ at 4 °C, concentrated by Nitrogen sweeping, and added 50 μL of MeOH was added for dissolution to the LC‐MS analysis.

##### Statistical Analysis

All statistical analysis was done by GraphPad Prism 8.

## Conflict of Interest

The authors declare no conflict of interest.

## Supporting information

Supplementary Material

## Data Availability

The data that support the findings of this study are available in the supplementary material of this article.
