# Supplementary material for: Design and Synthesis of Polyamine‐Proteolysis Targeting Chimera Conjugates for Histone Deacetylase (HDAC) Degradation with Enhanced Cellular Uptake
Source: ChemistryOpen. 2025 Nov 2;15(4):e202500356. doi: 10.1002/open.202500356 (PMC13054233; doi:10.1002/open.202500356)

Supporting Information

Design and Synthesis of Polyamine-PROTAC Conjugates for Histone Deacetylase (HDAC) Degradation with Enhanced Cellular Uptake

Yanran Liu,[a] # Wentian Chen,[a] # Yanwei Shang,[a] Chaonan Tang,[a] Xianming Zeng,[a] Jun Li,*[a] Wenting Du ,*[a]

[a] Hangzhou Medical College, Hangzhou, 311399, P. R. China;

* Corresponding author Email: W. Du: ddwwtt@163.com; J. Li: lijun@hmc.edu.cn

Content

1. The Copies of ^{1}H NMR and ^{13}C NMR Spectrum S1

1. The Copies of ^{1}H NMR and ^{13}C NMR Spectrum

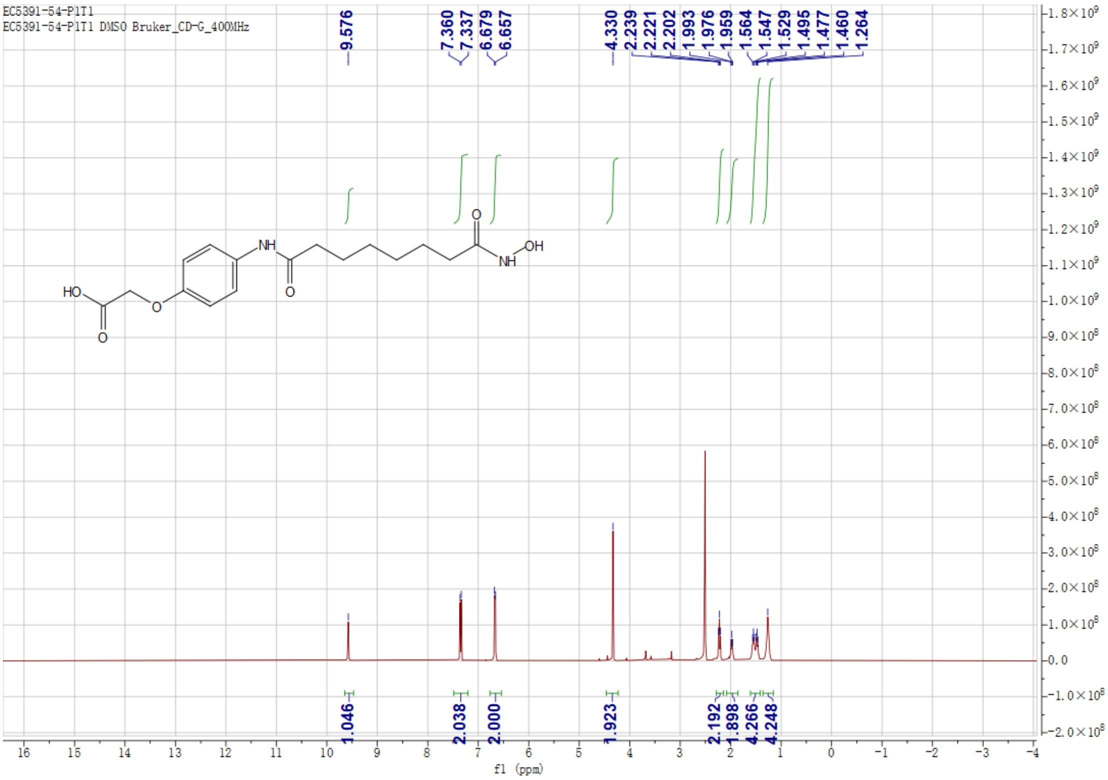

^{1}H NMR of **5**

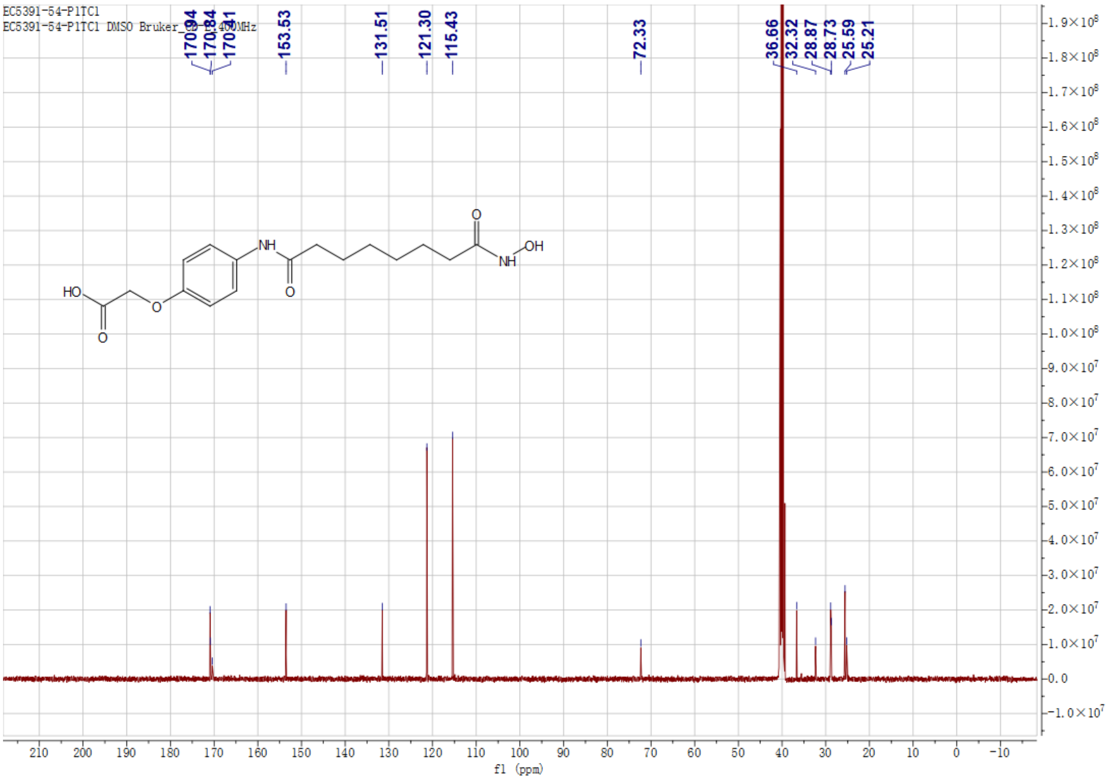

^{13}C NMR of **5**

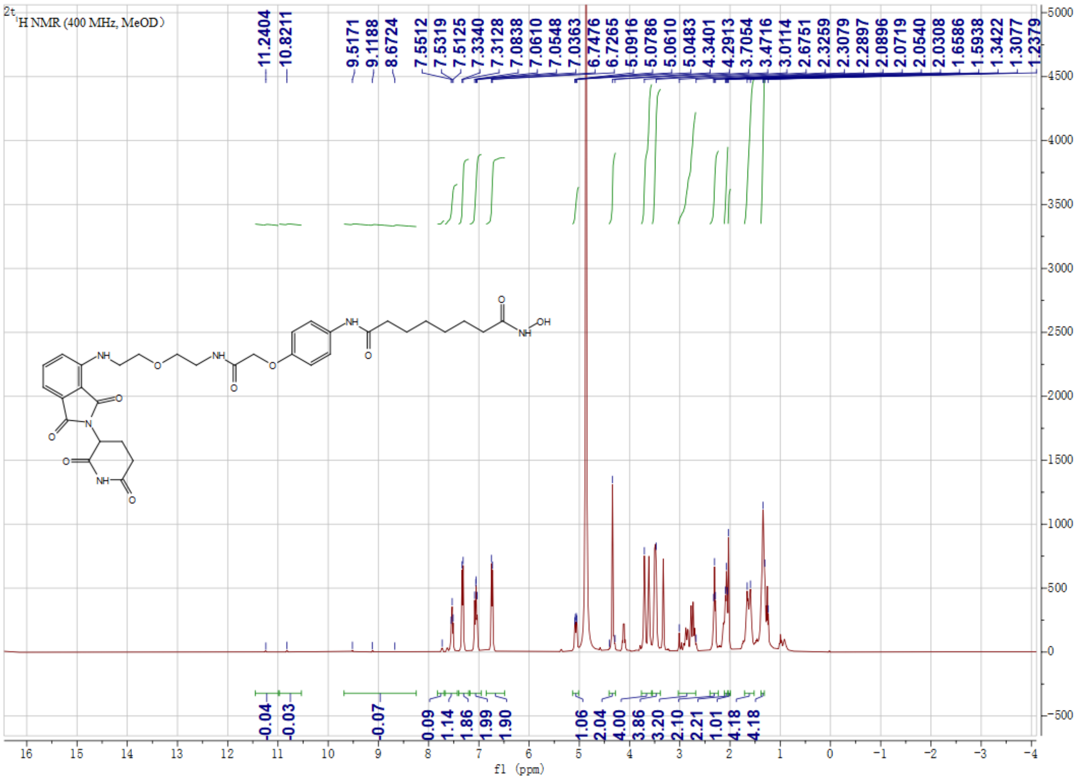

^{1}H NMR of **A**

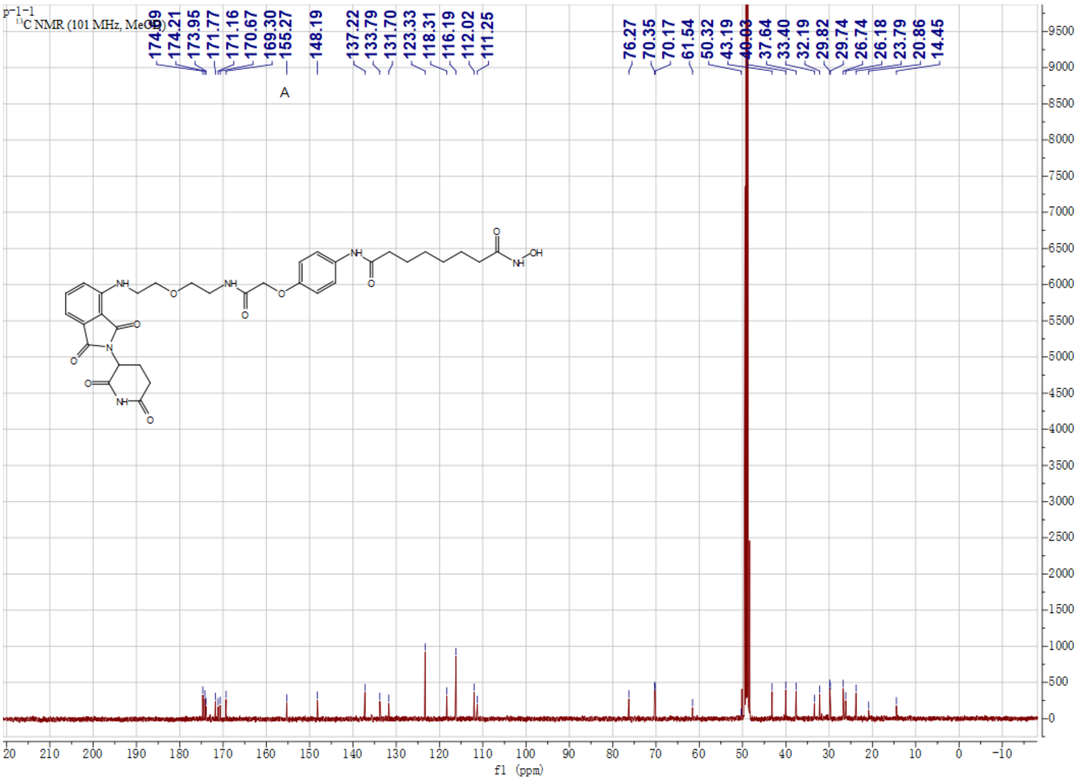

^{13}C NMR of **A**

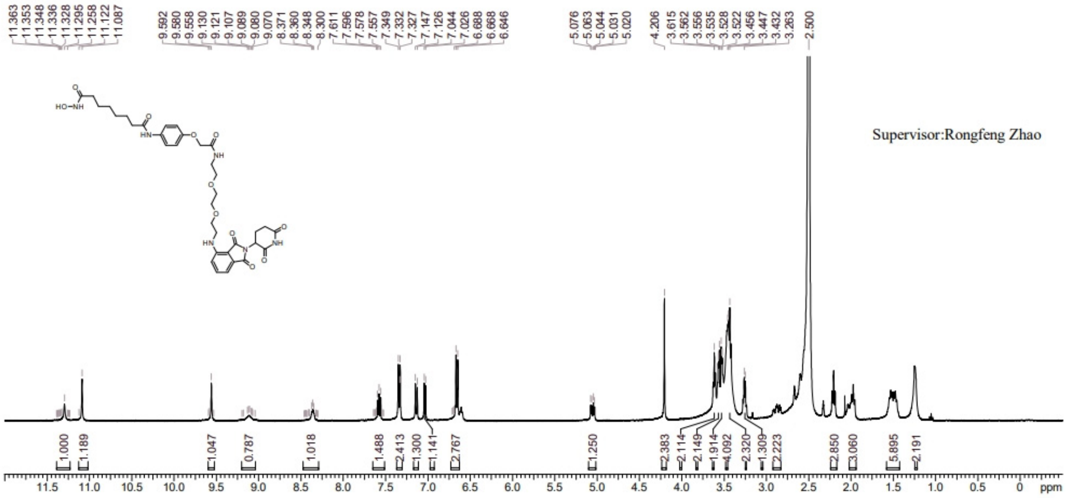

^{1}H NMR of **B**

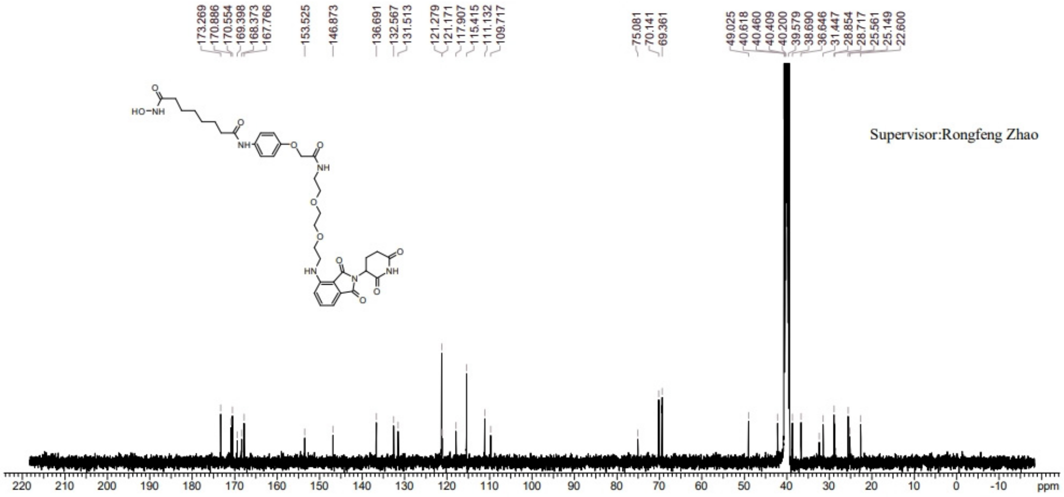

^{13}C NMR of **B**

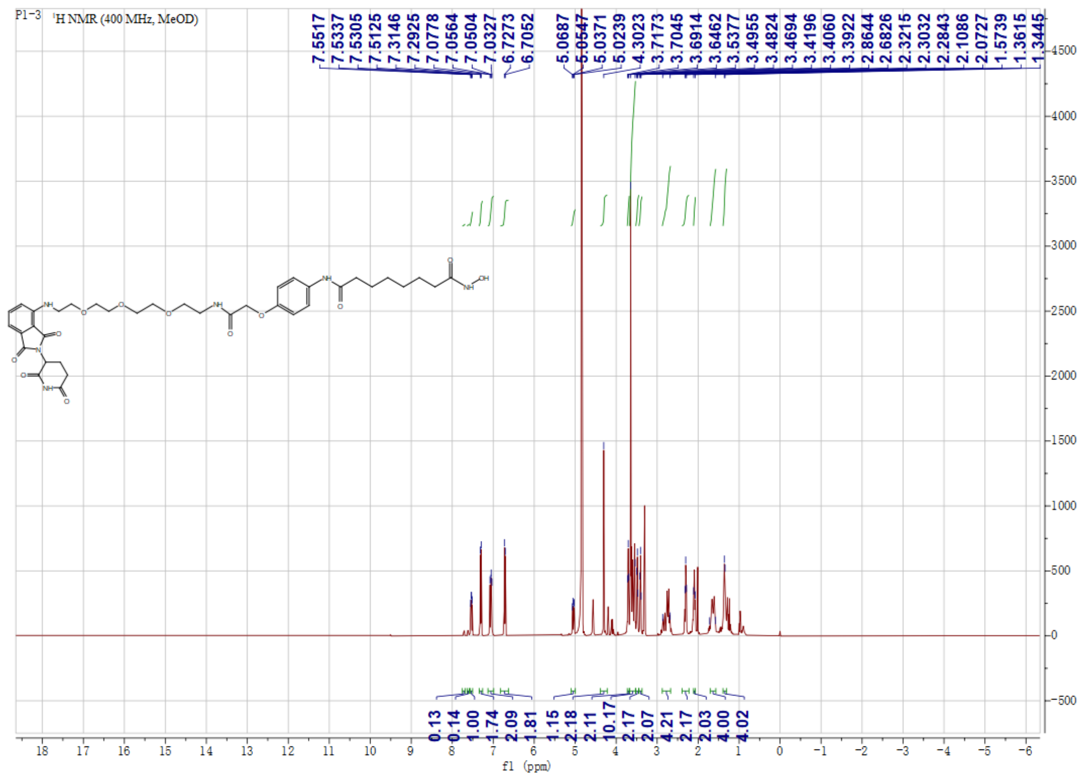

^{1}H NMR of **C**

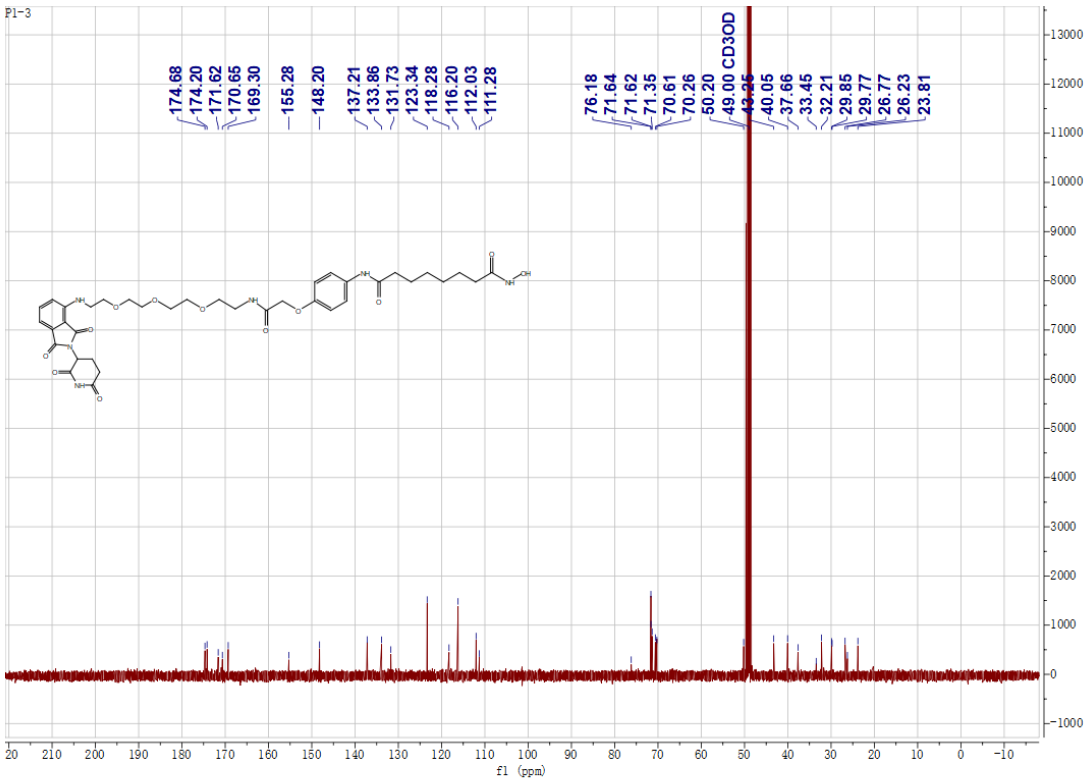

^{13}C NMR of **C**

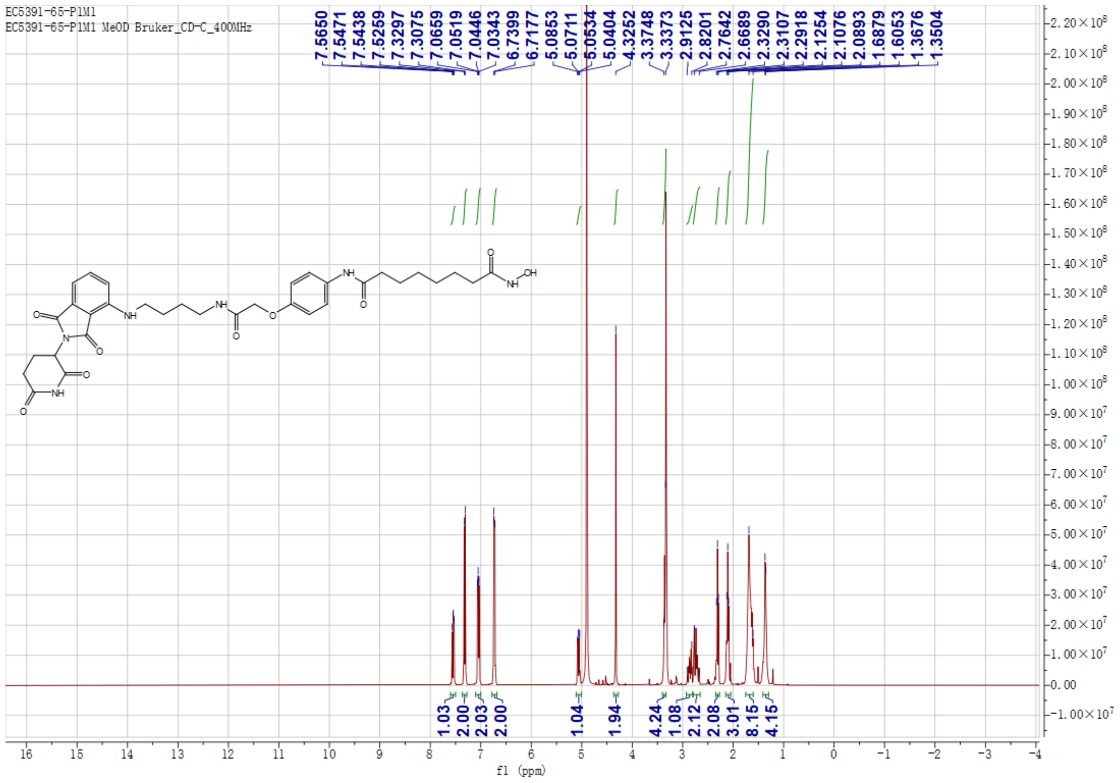

^{1}H NMR of **D**

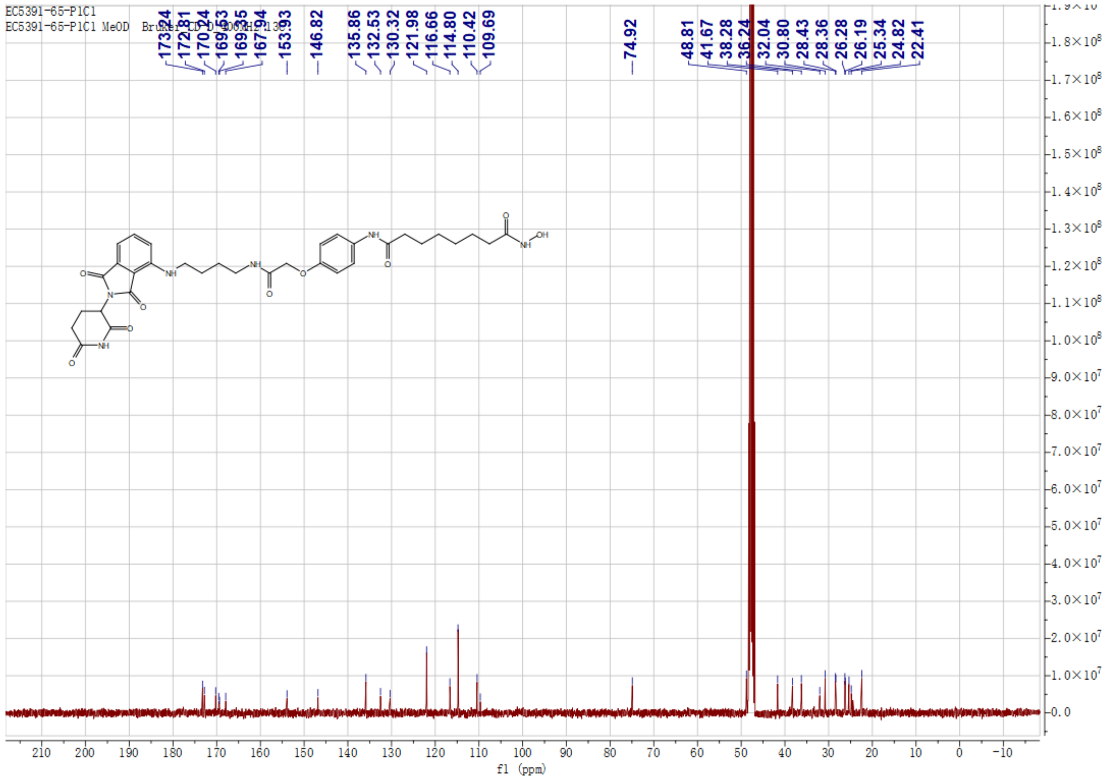

^{13}C NMR of **D**

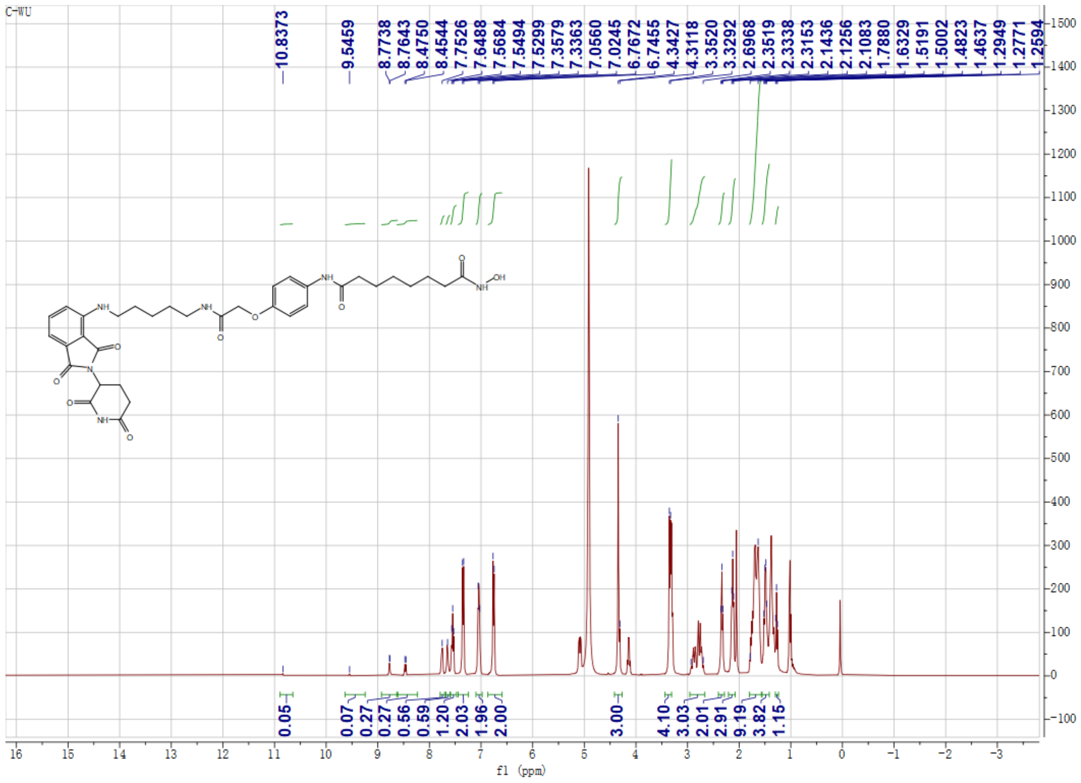

^{1}H NMR of **E**

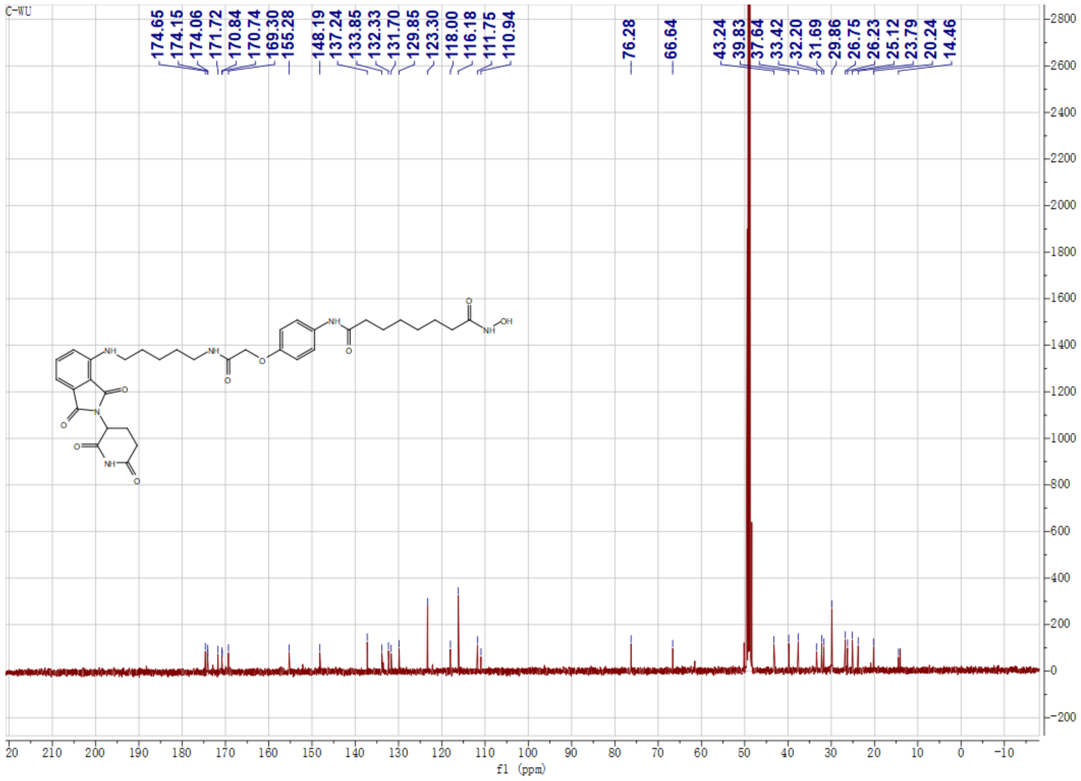

^{13}C NMR of **E**

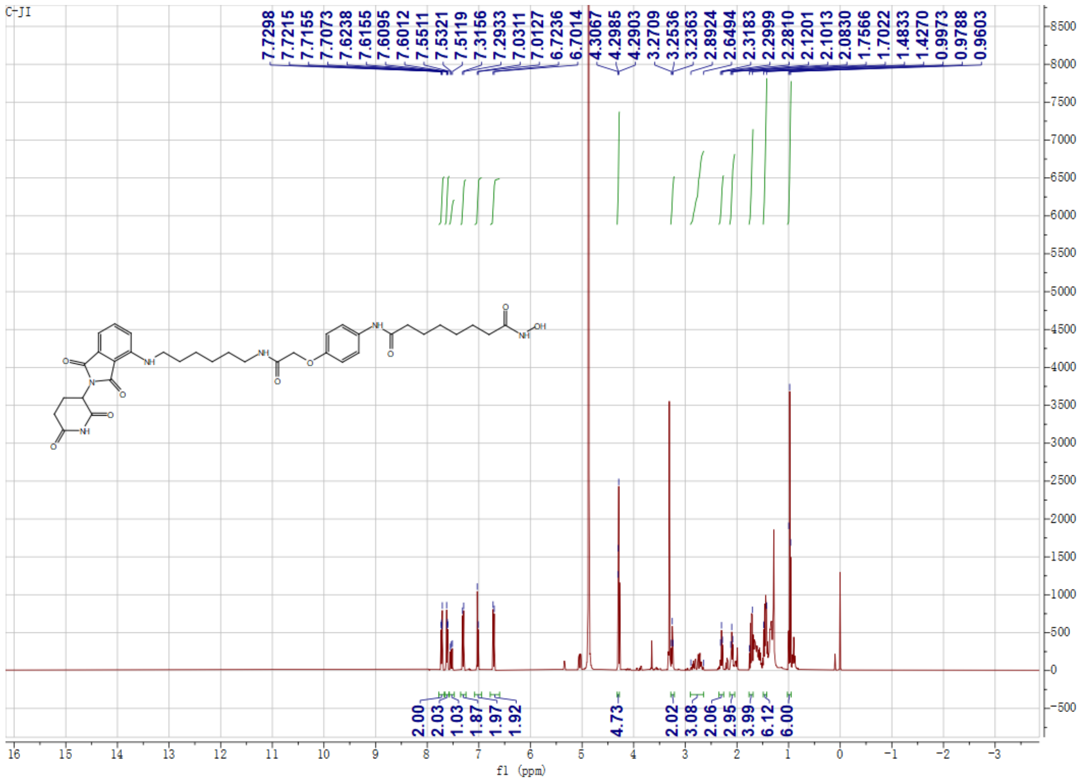

^{1}H NMR of **F**

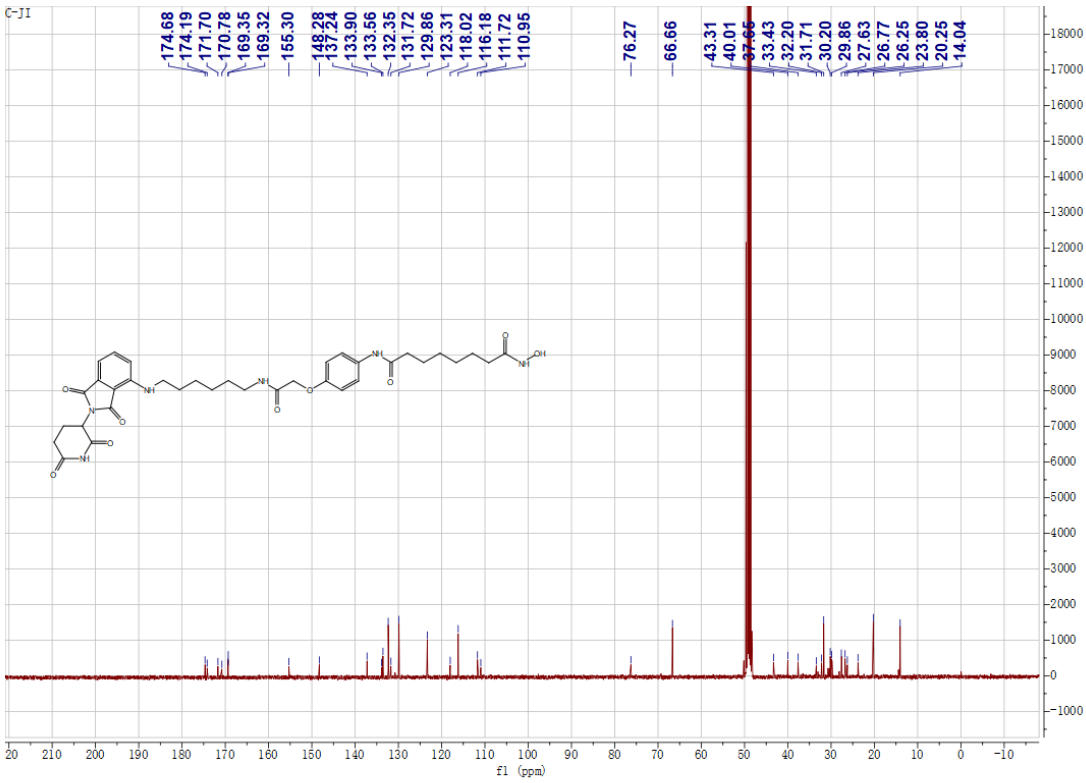

^{13}C NMR of **F**

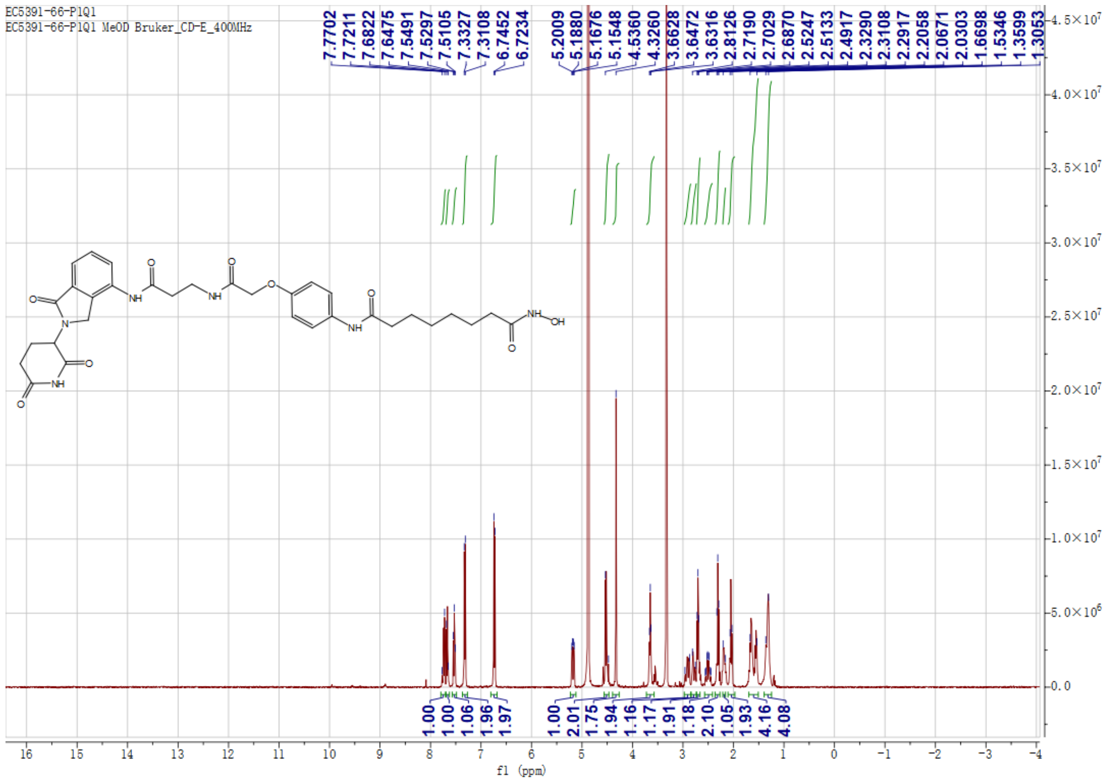

^{1}H NMR of **G**

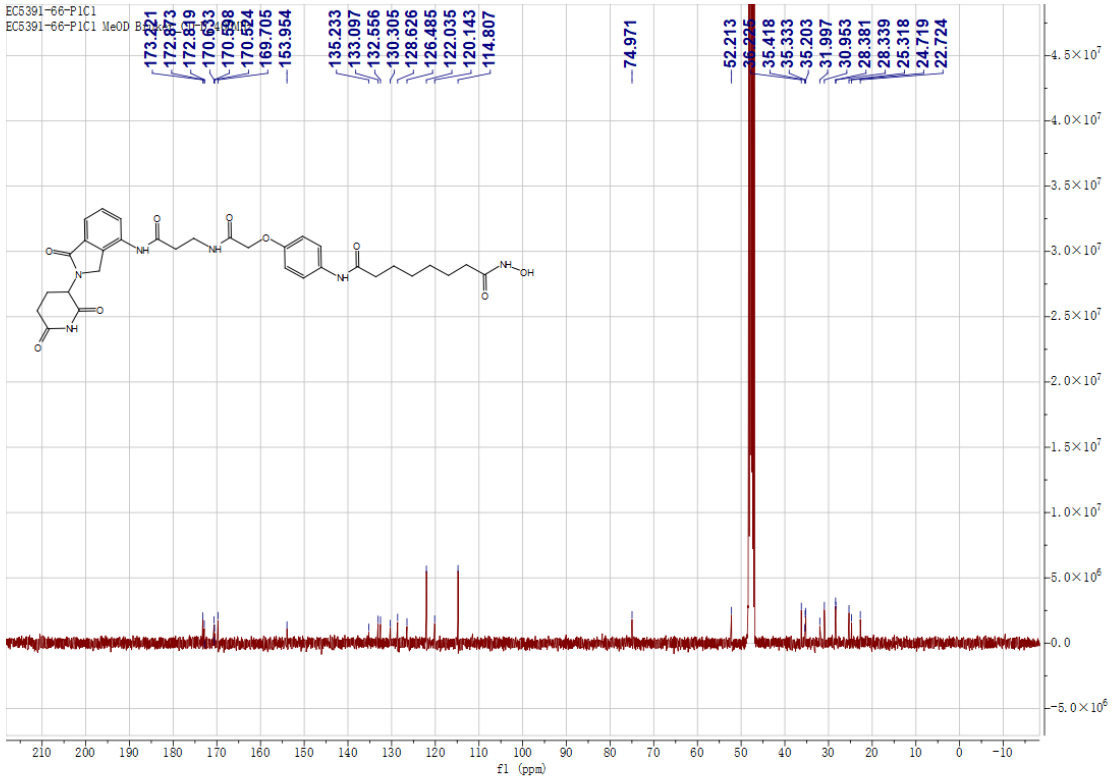

^{13}C NMR of **G**

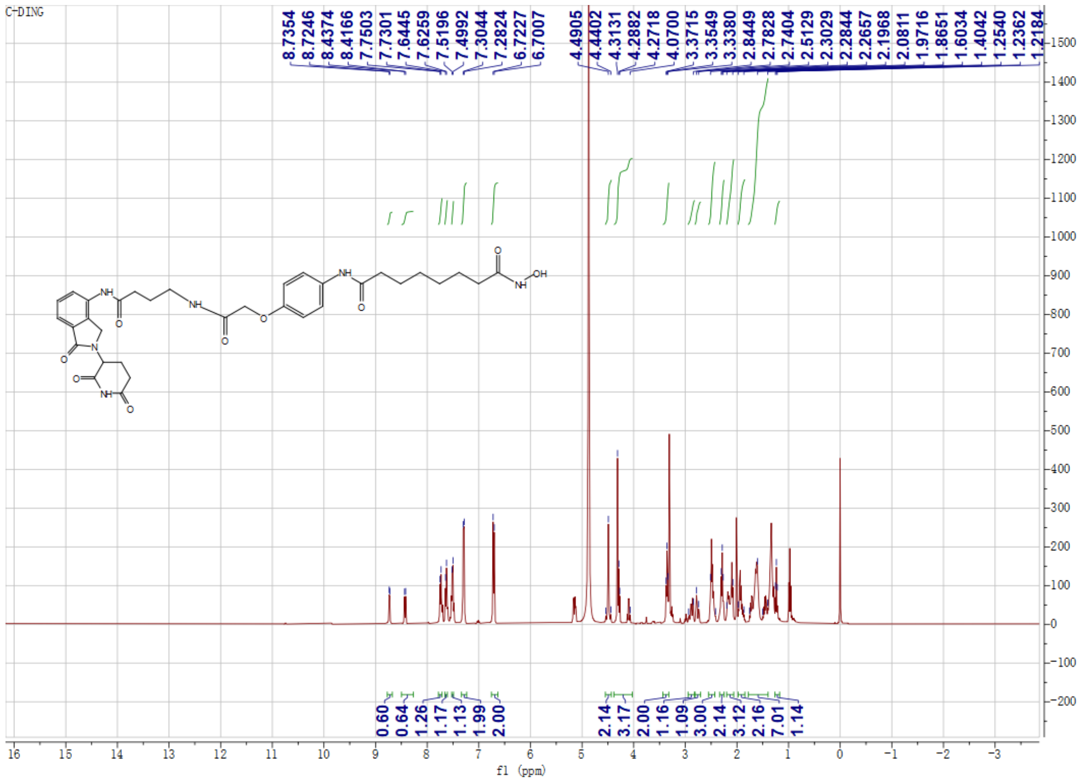

^{1}H NMR of **H**

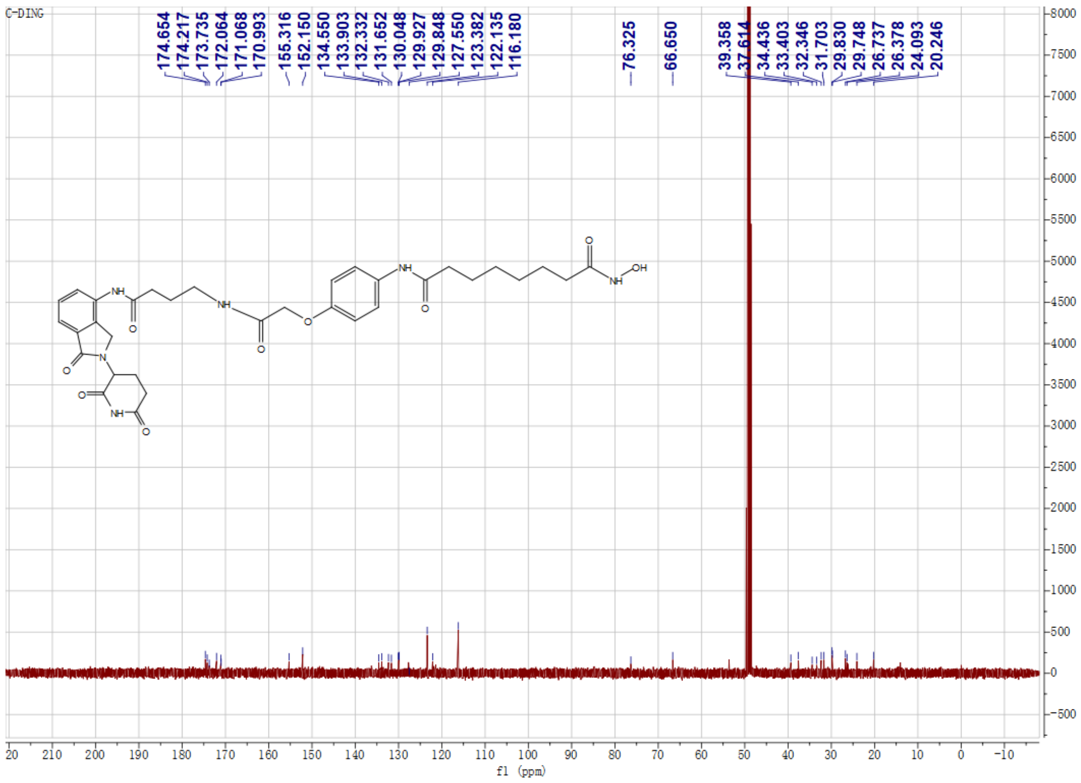

^{13}C NMR of **H**

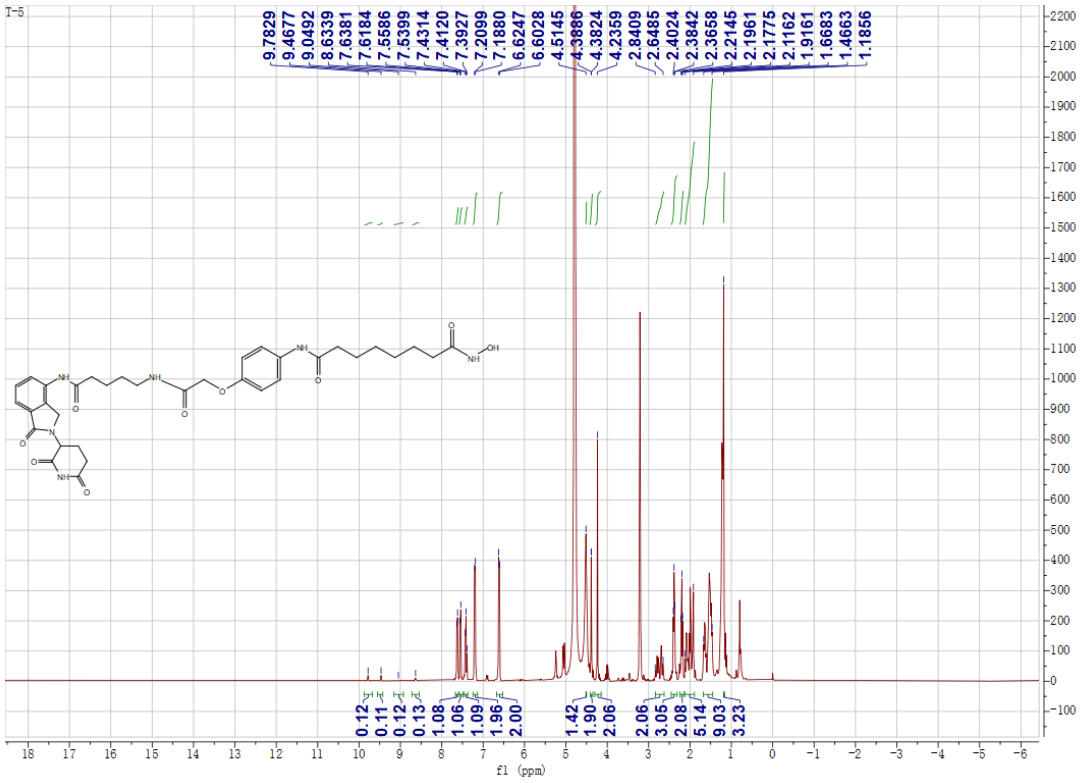

^{1}H NMR of **I**

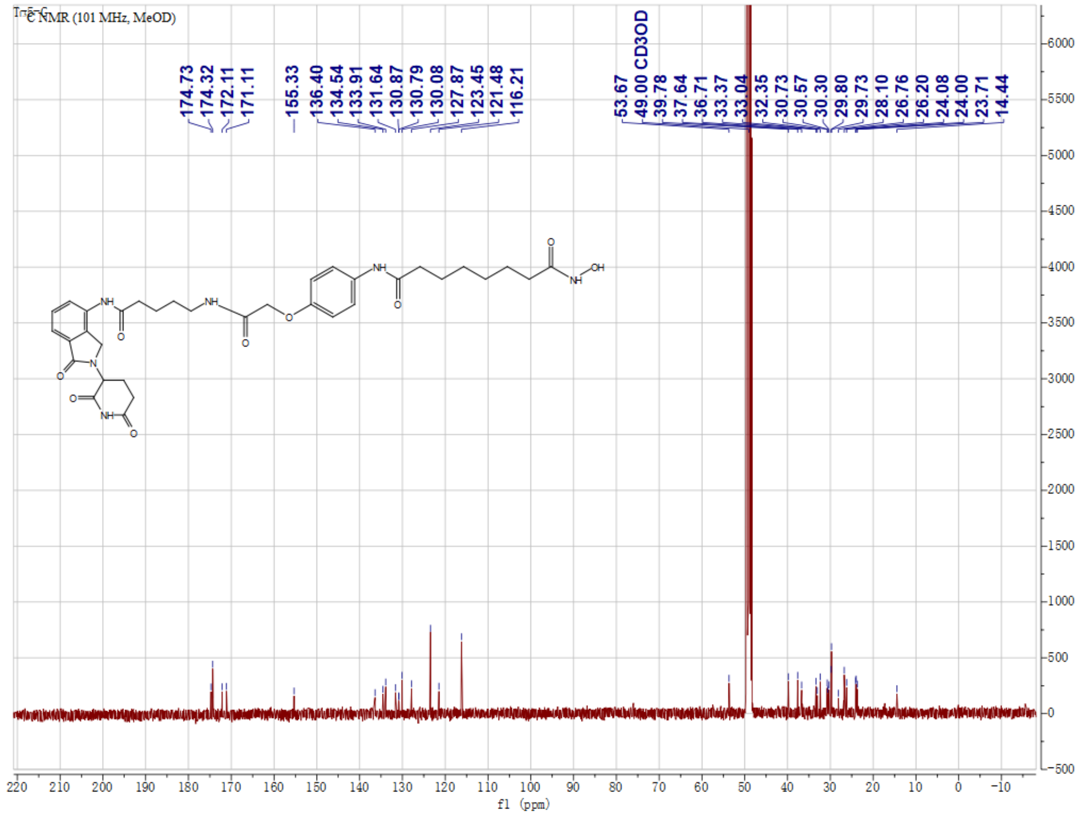

^{13}C NMR of **I**

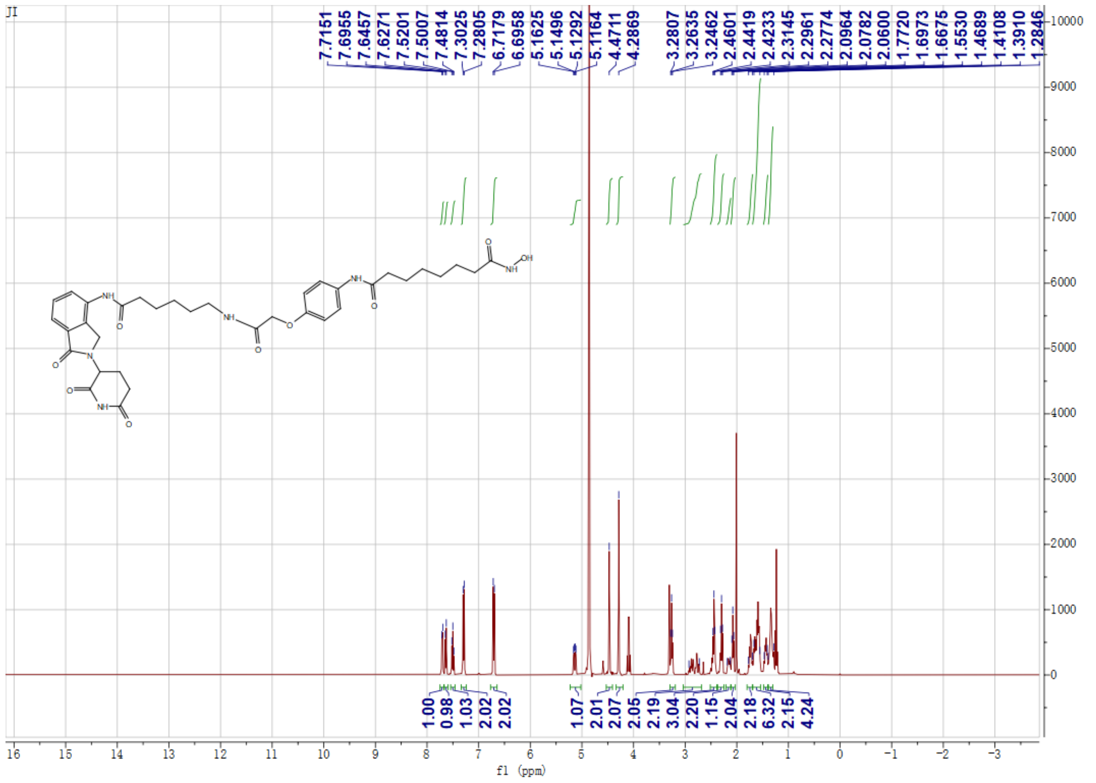

^{1}H NMR of **J**

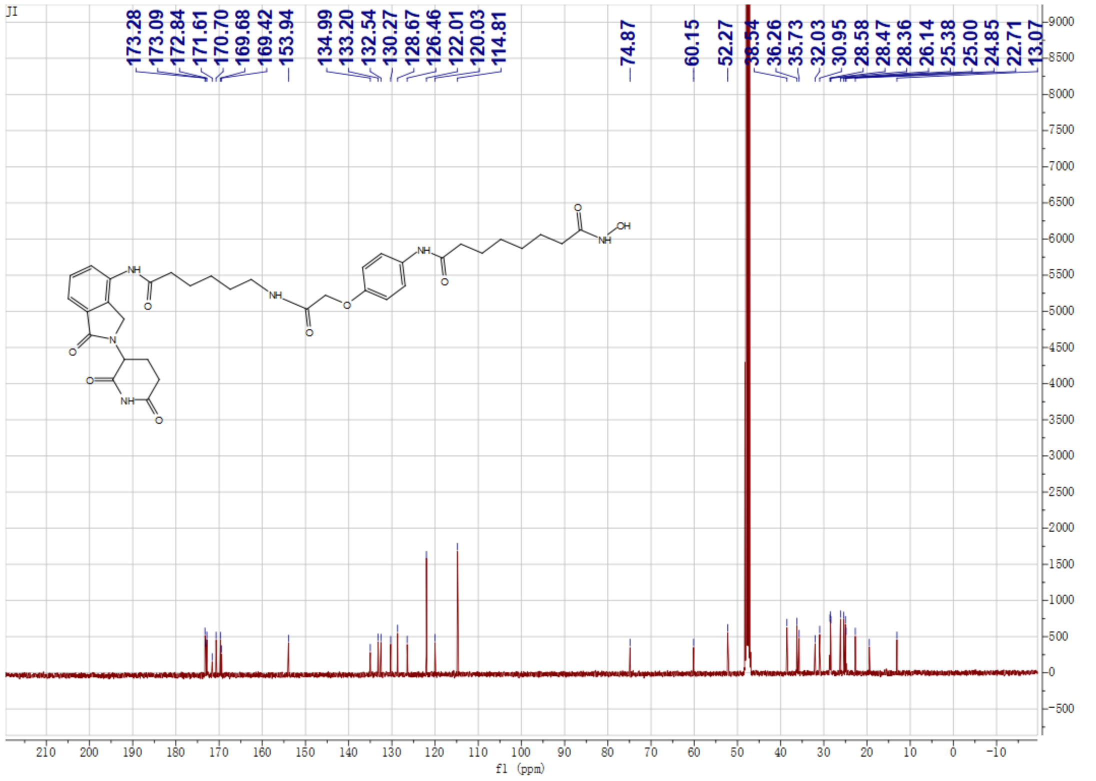

^{13}C NMR of **J**

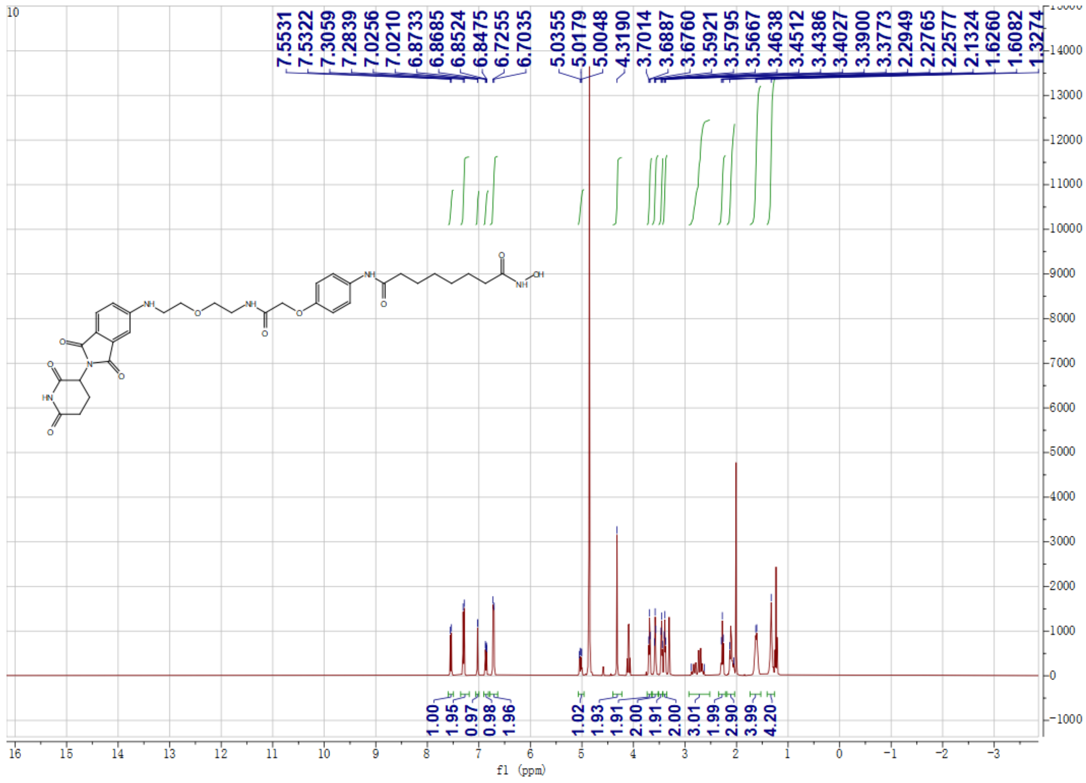

^{1}H NMR of **K**

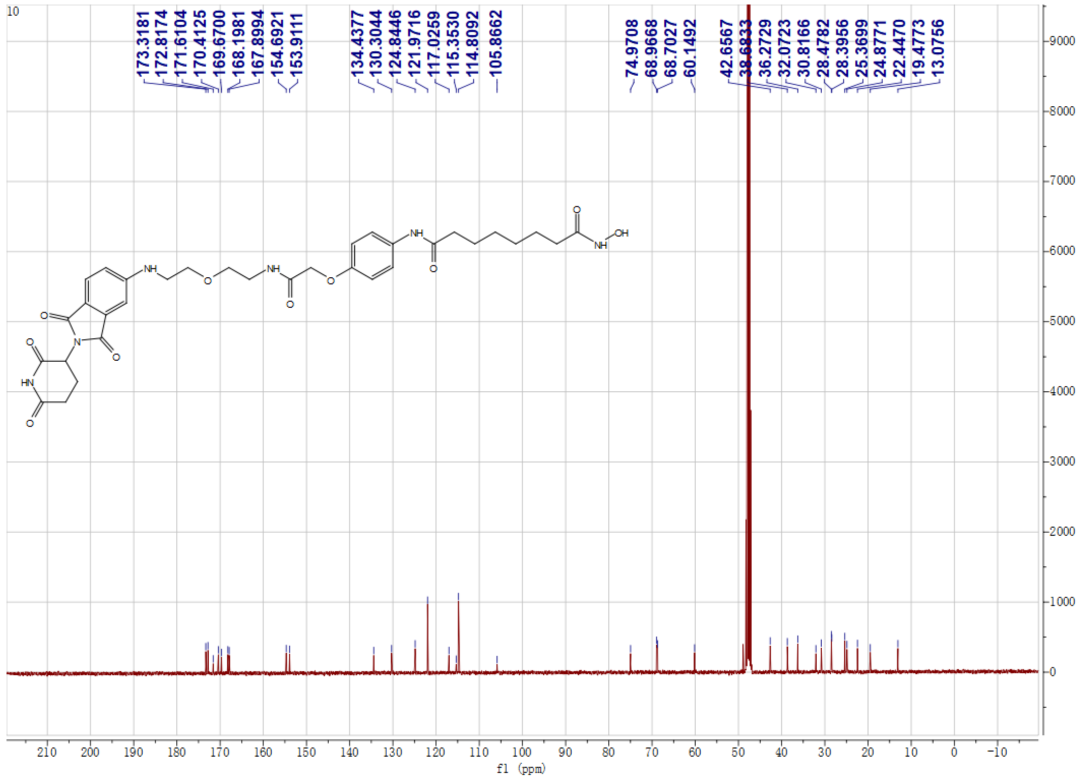

^{13}C NMR of **K**

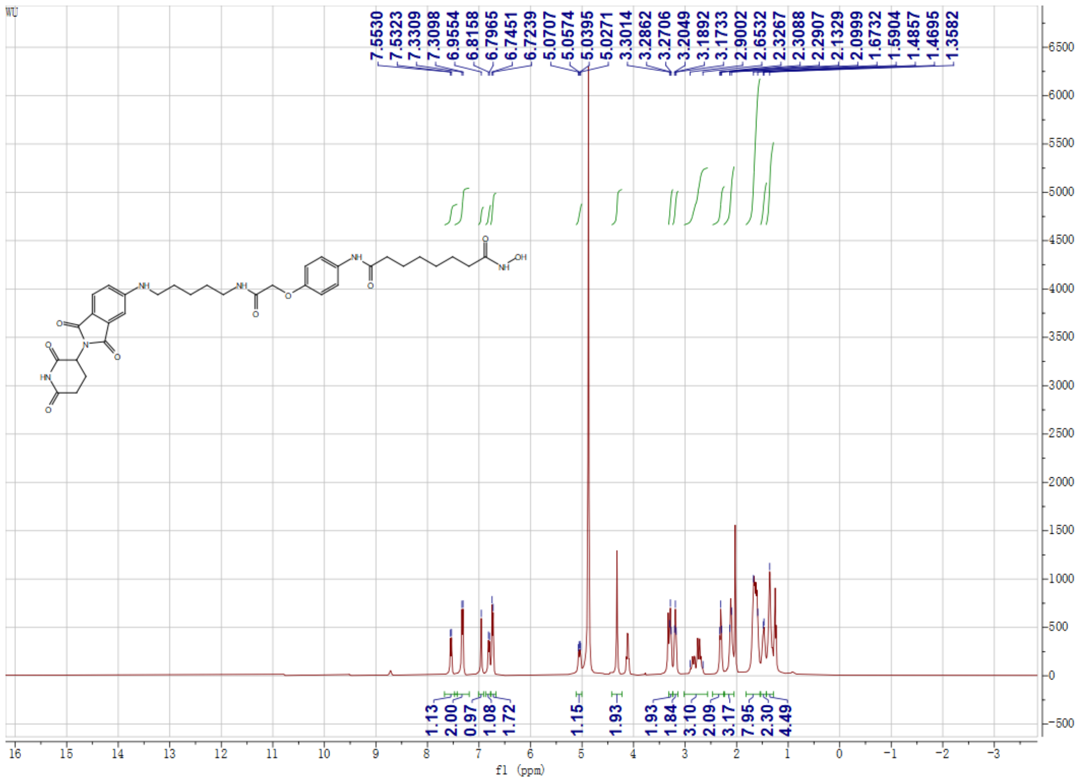

^{1}H NMR of **L**

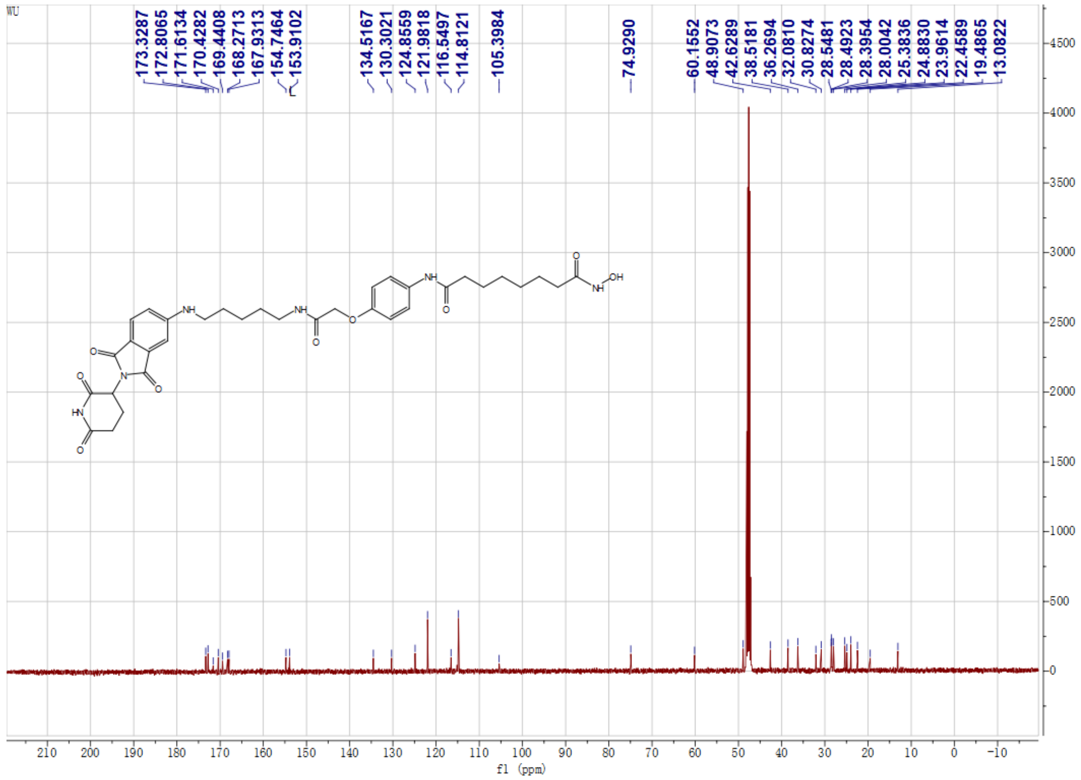

^{13}C NMR of **L**

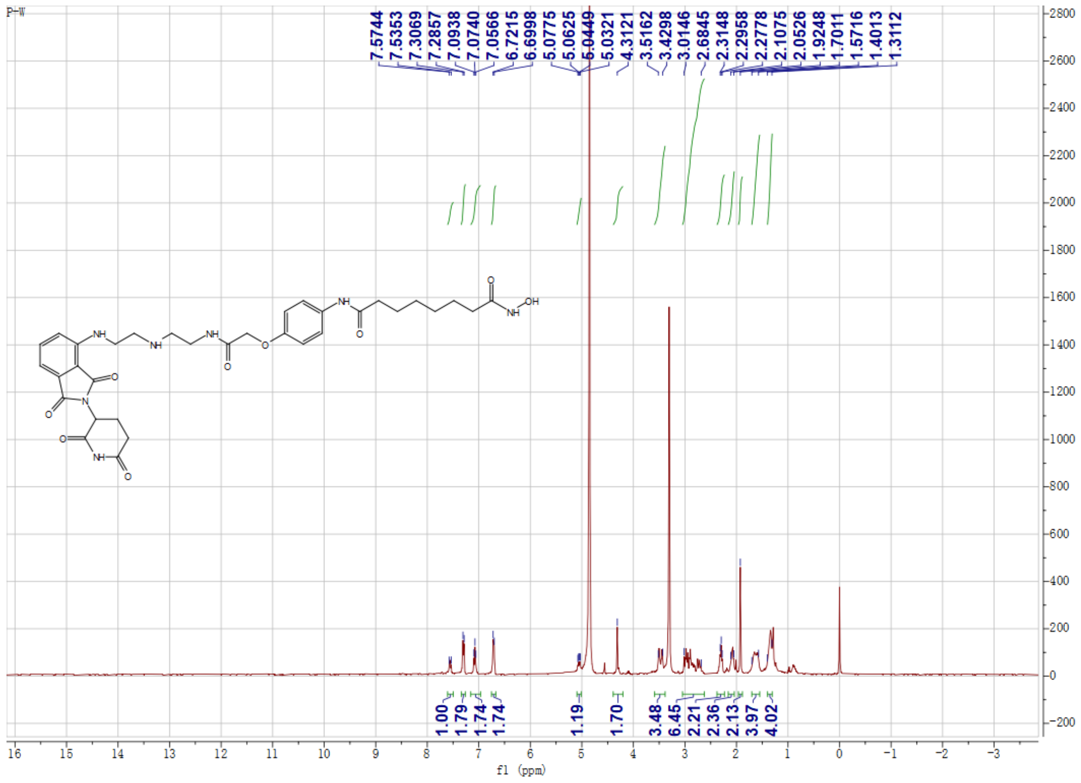

^{1}H NMR of **M**

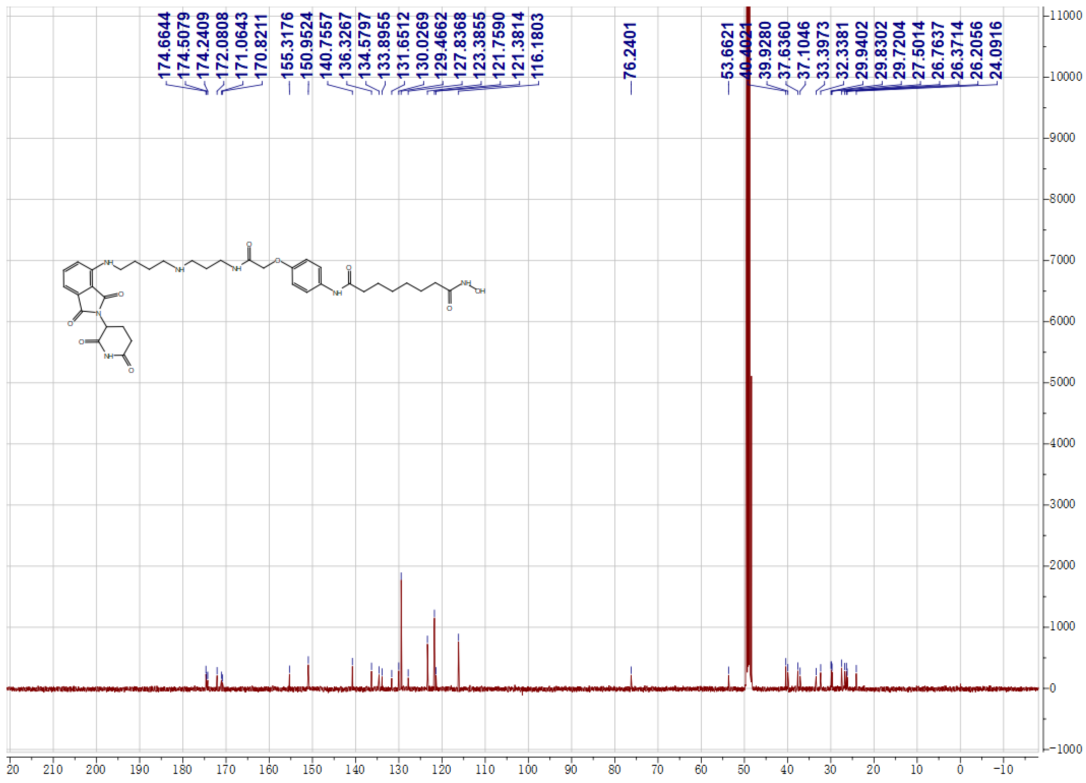

^{13}C NMR of **M**

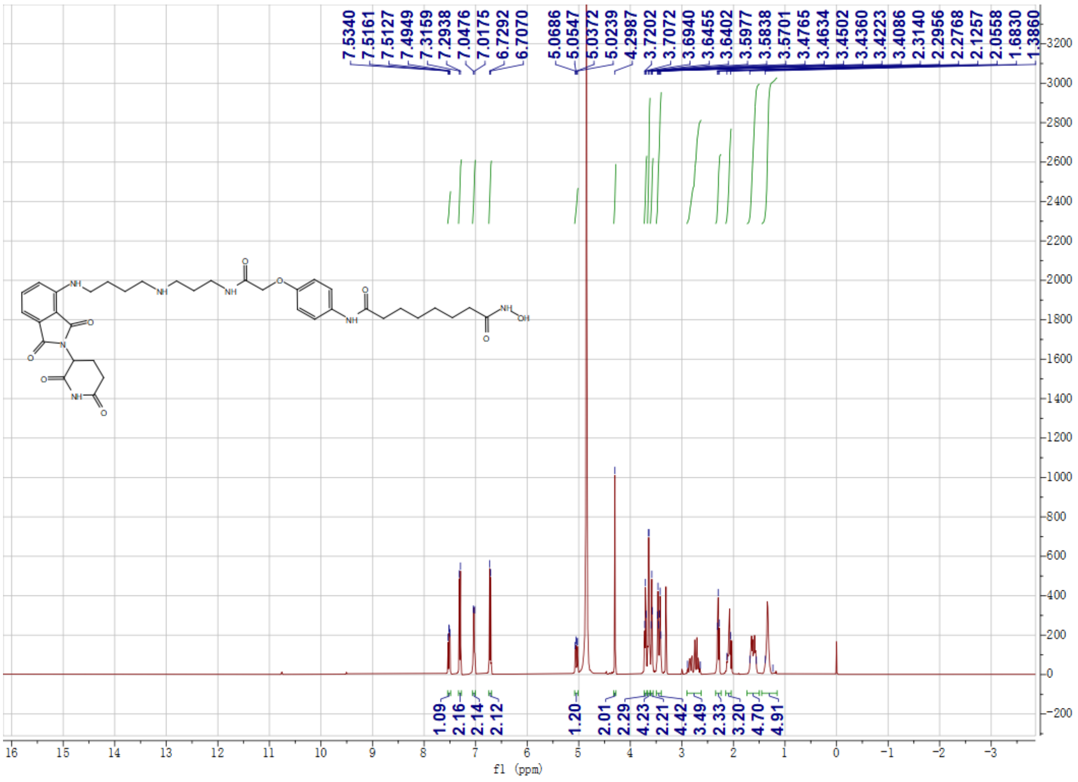

^{1}H NMR of **N**

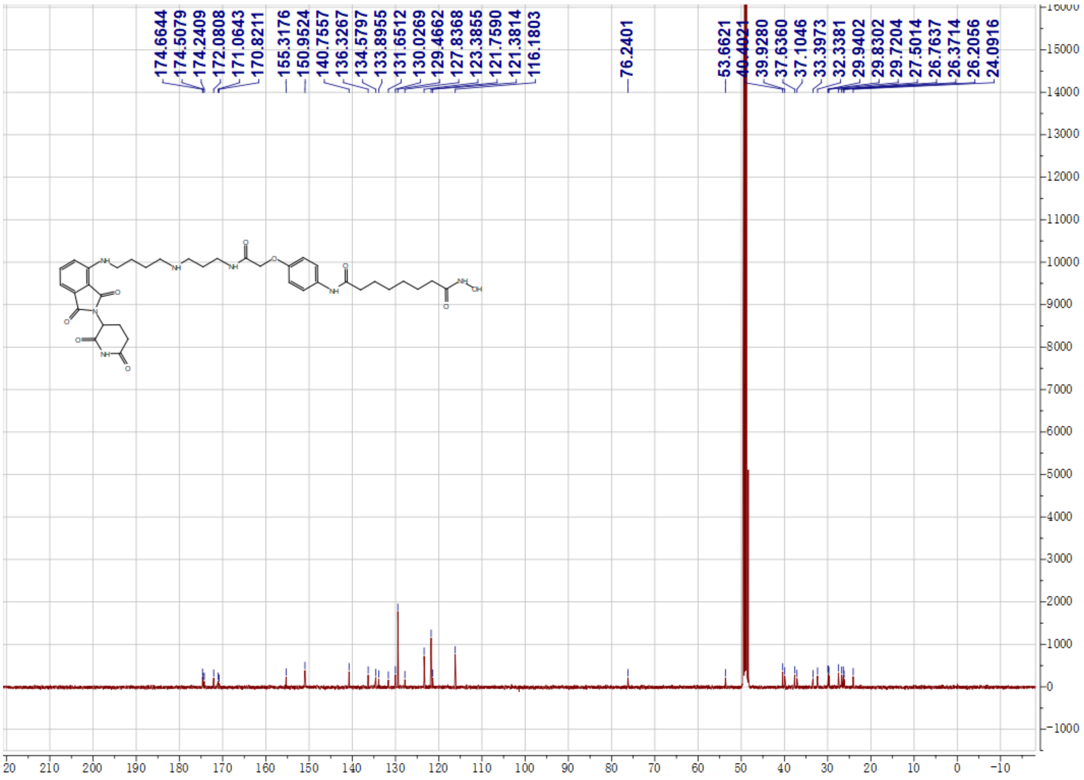

^{13}C NMR of **N**

3. MS spectrum

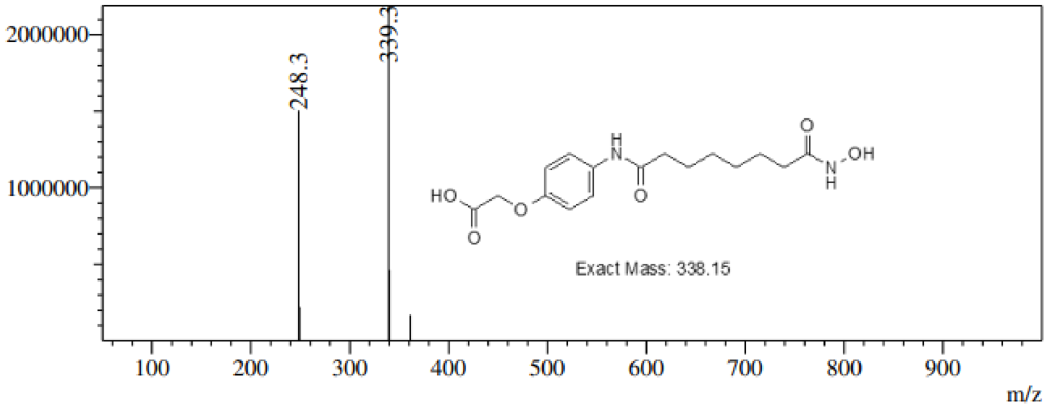

2000000
1000000
248.3
339.3
HO
O
O
H
N
O
O
N
OH
H
Exact Mass: 338.15
100
200
300
400
500
600
700
800
900
m/z

MS of **5**

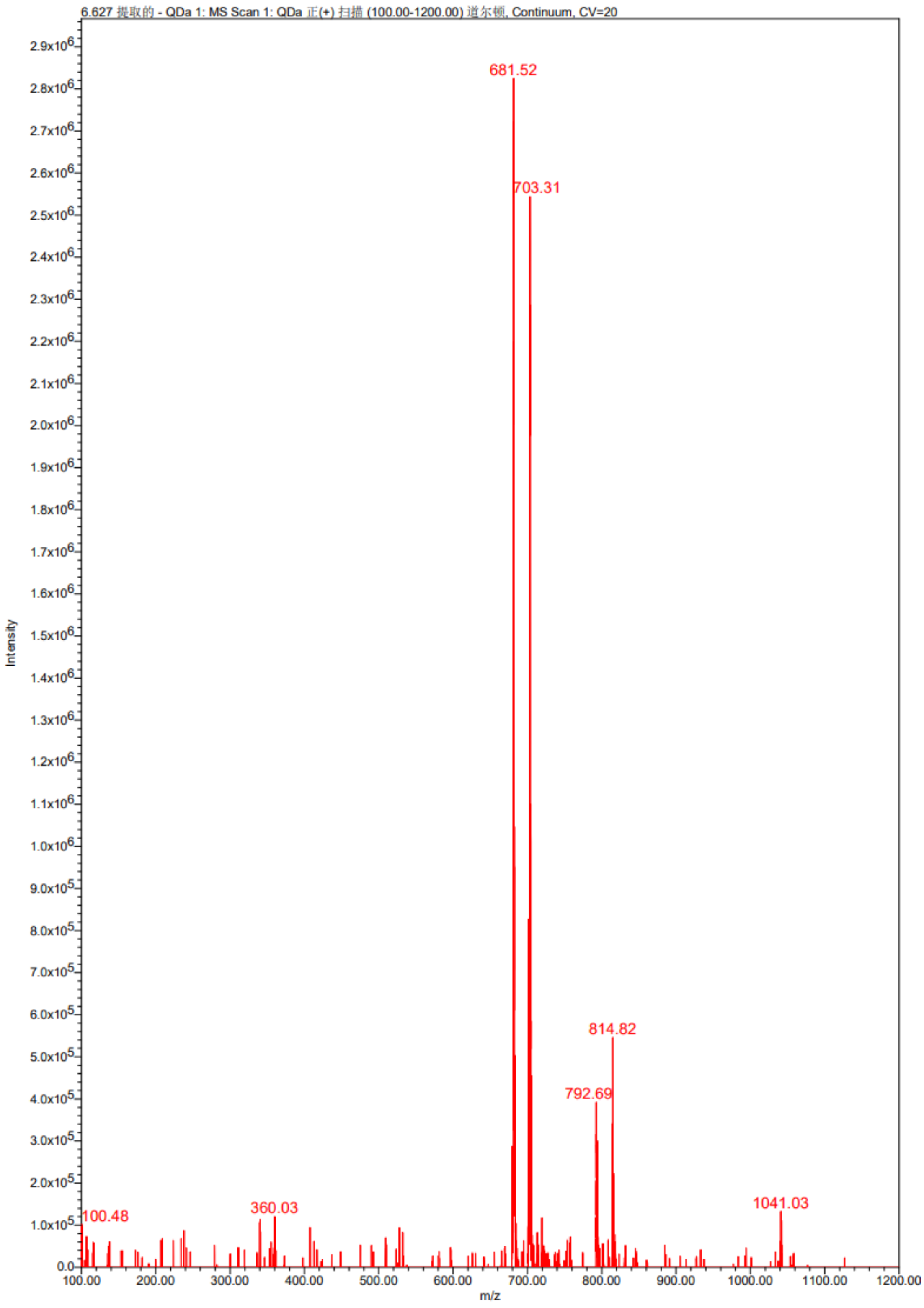
6.627 提取的 - QDa 1: MS Scan 1: QDa 正(+) 扫描 (100.00-1200.00) 道尔顿, Continuum, CV=20
Intensity
m/z
681.52
703.31
814.82
792.69
1041.03
360.03
100.48

MS of **A**

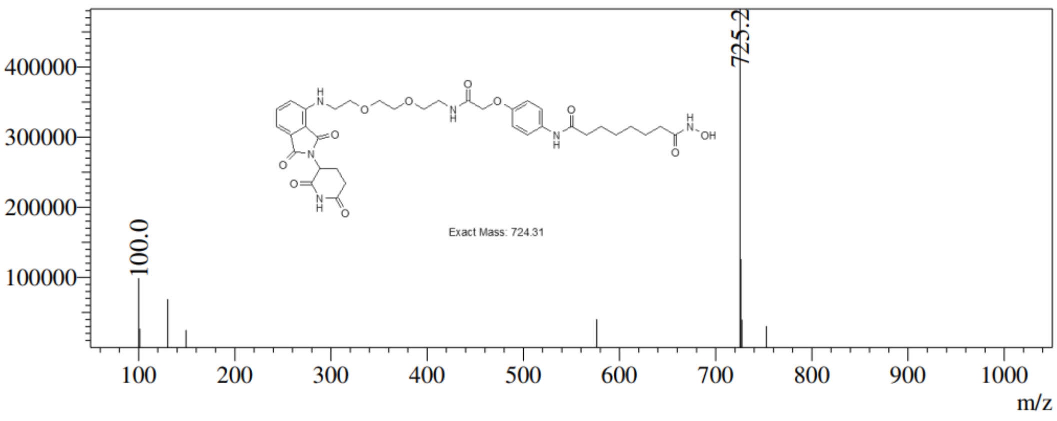
725.2
100.0
400000
300000
200000
100000
100
200
300
400
500
600
700
800
900
1000
m/z
Exact Mass: 724.31

MS of **B**

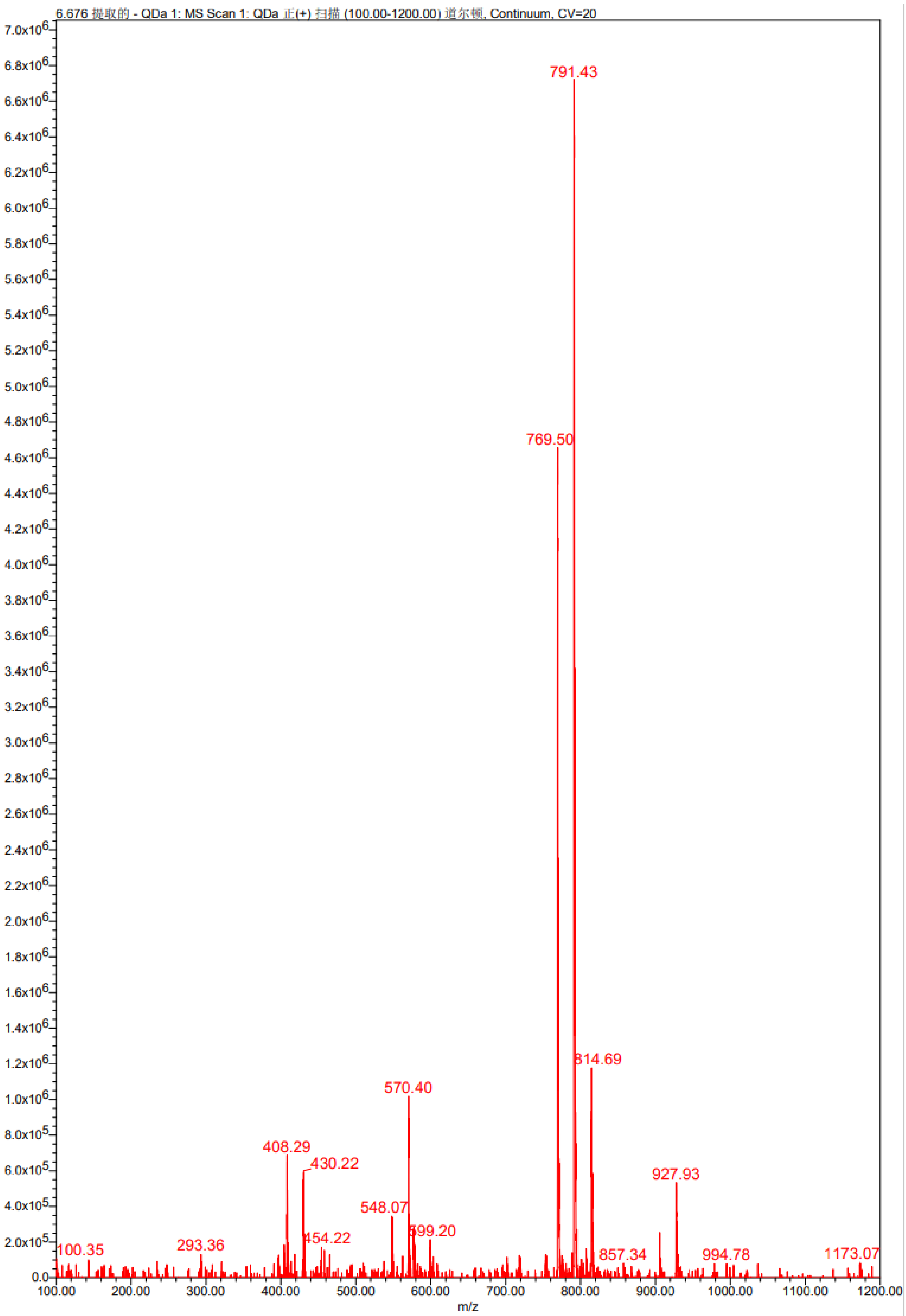
6.676 提取的 - QDa 1: MS Scan 1: QDa 正(+) 扫描 (100.00-1200.00) 道尔顿, Continuum, CV=20
791.43
769.50
814.69
570.40
408.29
430.22
927.93
548.07
599.20
454.22
100.35
293.36
857.34
994.78
1173.07
m/z

MS of **C**

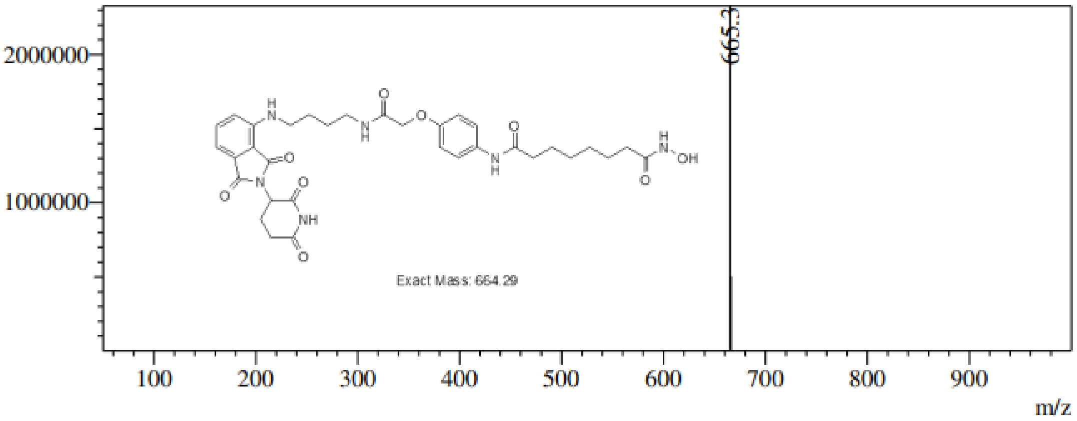
2000000
1000000
665.3
H
N
O
O
N
H
O
O
N
H
O
H
N
OH
O
O
N
O
NH
O
Exact Mass: 664.29
100
200
300
400
500
600
700
800
900
m/z

MS of **D**

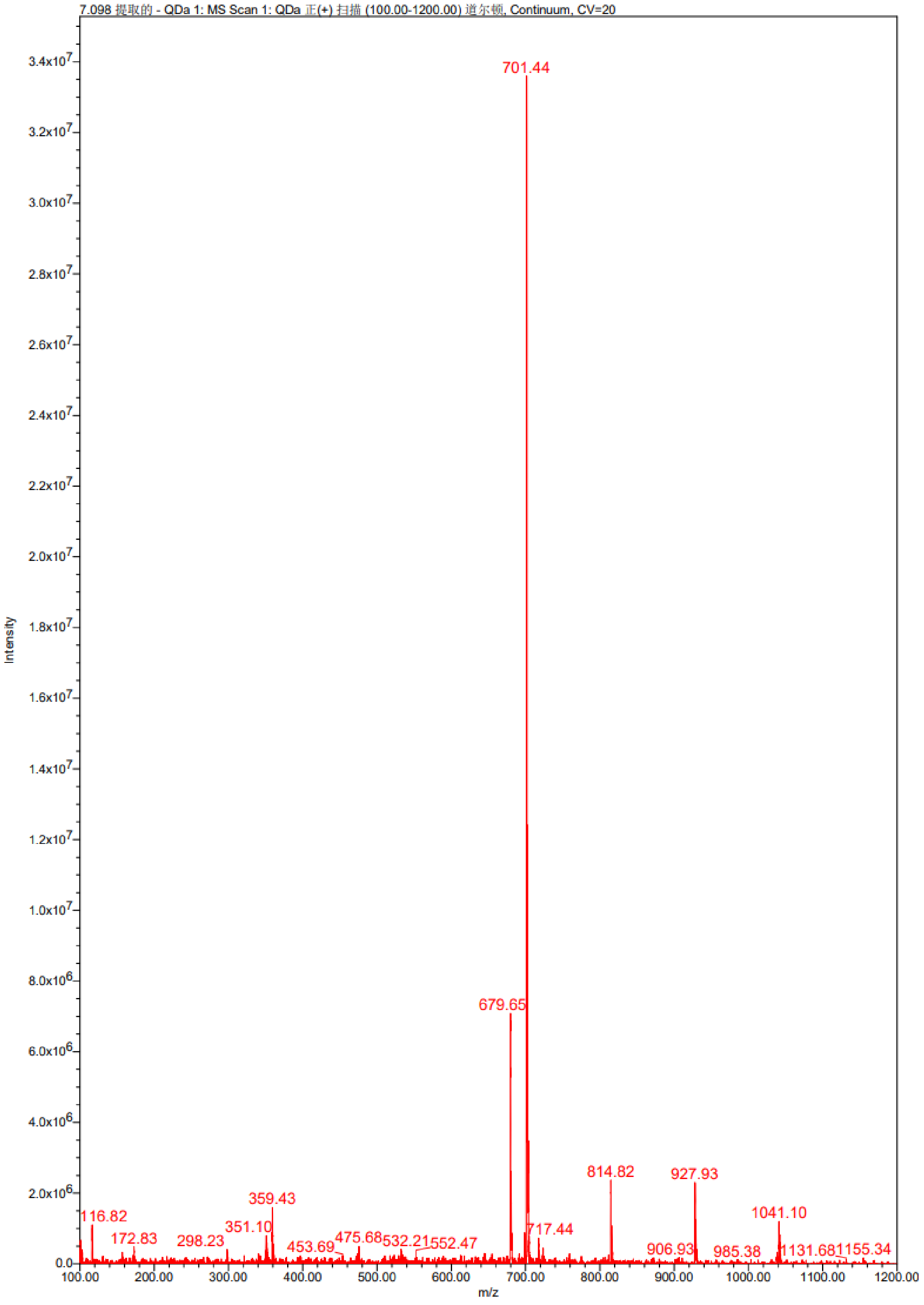

7.098 提取的 - QDa 1: MS Scan 1: QDa 正(+) 扫描 (100.00-1200.00) 道尔顿, Continuum, CV=20
Intensity
m/z
701.44
679.65
814.82
927.93
359.43
1041.10
116.82
351.10
717.44
172.83
298.23
475.68
532.21
552.47
453.69
906.93
985.38
1131.68
1155.34

MS of **E**

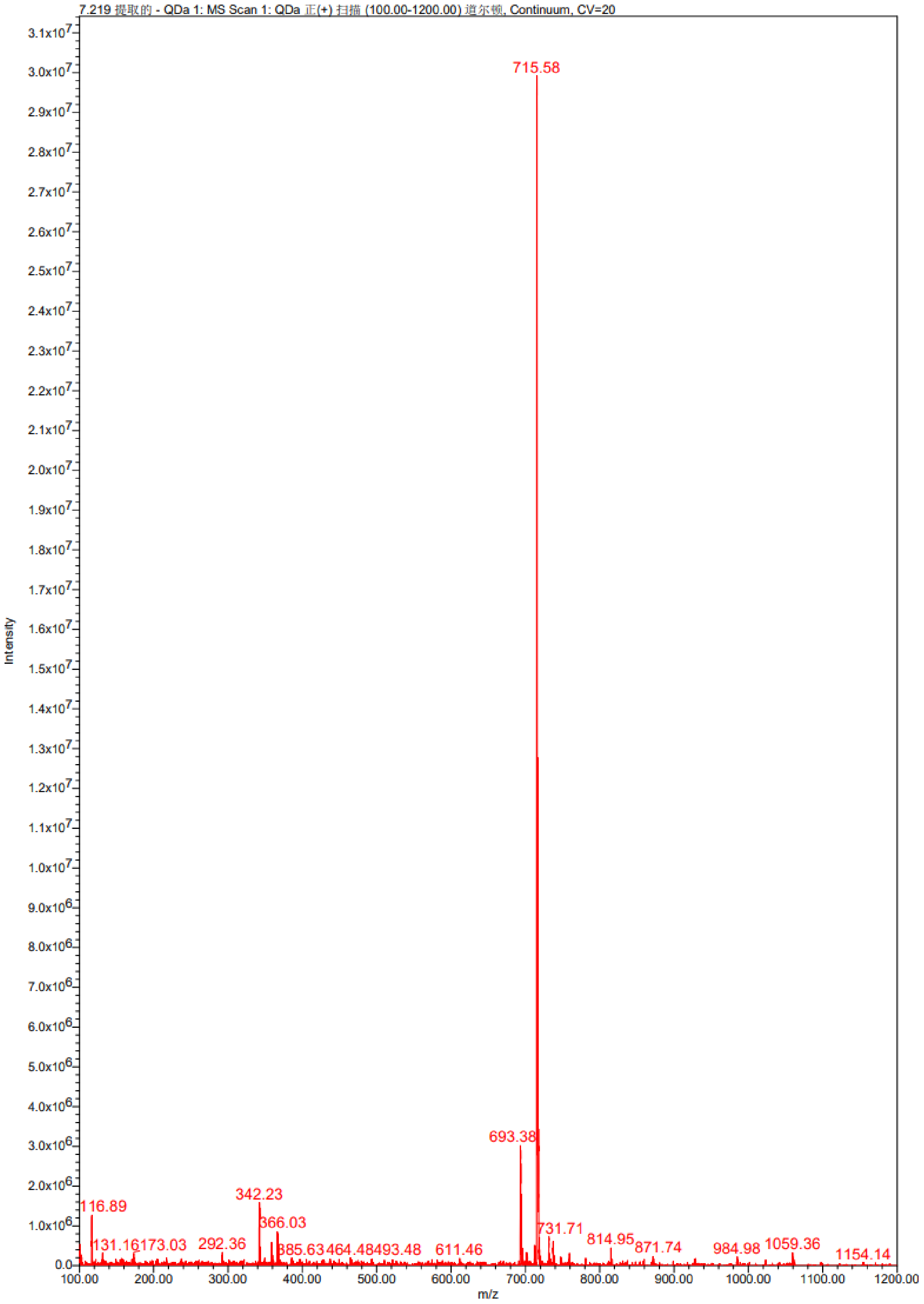
7.219 提取的 - QDa 1: MS Scan 1: QDa 正(+) 扫描 (100.00-1200.00) 道尔顿, Continuum, CV=20
Intensity
m/z
715.58
693.38
342.23
116.89
366.03
731.71
814.95
871.74
984.98
1059.36
1154.14
131.16
173.03
292.36
385.63
464.48
493.48
611.46

MS of **F**

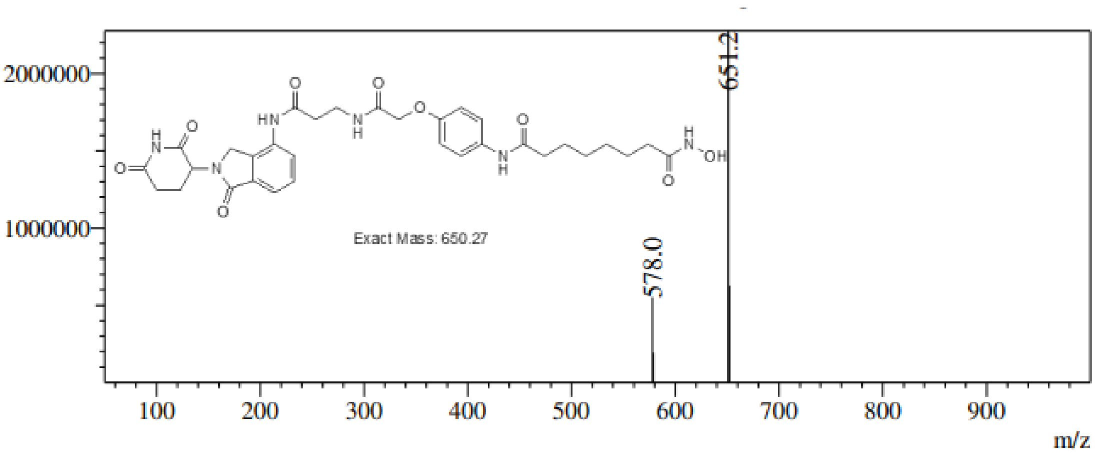

2000000
1000000
Exact Mass: 650.27
578.0
651.2
100
200
300
400
500
600
700
800
900
m/z

MS of **G**

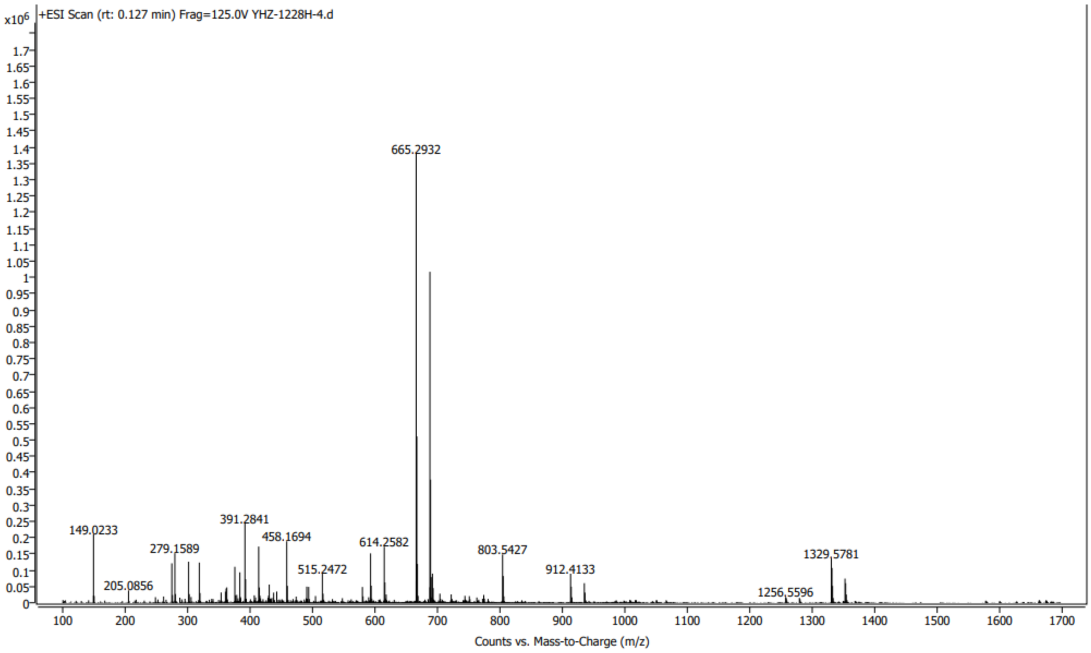

x10^6
+ESI Scan (rt: 0.127 min) Frag=125.0V YHZ-1228H-4.d
665.2932
149.0233
205.0856
279.1589
391.2841
458.1694
515.2472
614.2582
803.5427
912.4133
1256.5596
1329.5781
Counts vs. Mass-to-Charge (m/z)

MS of **H**

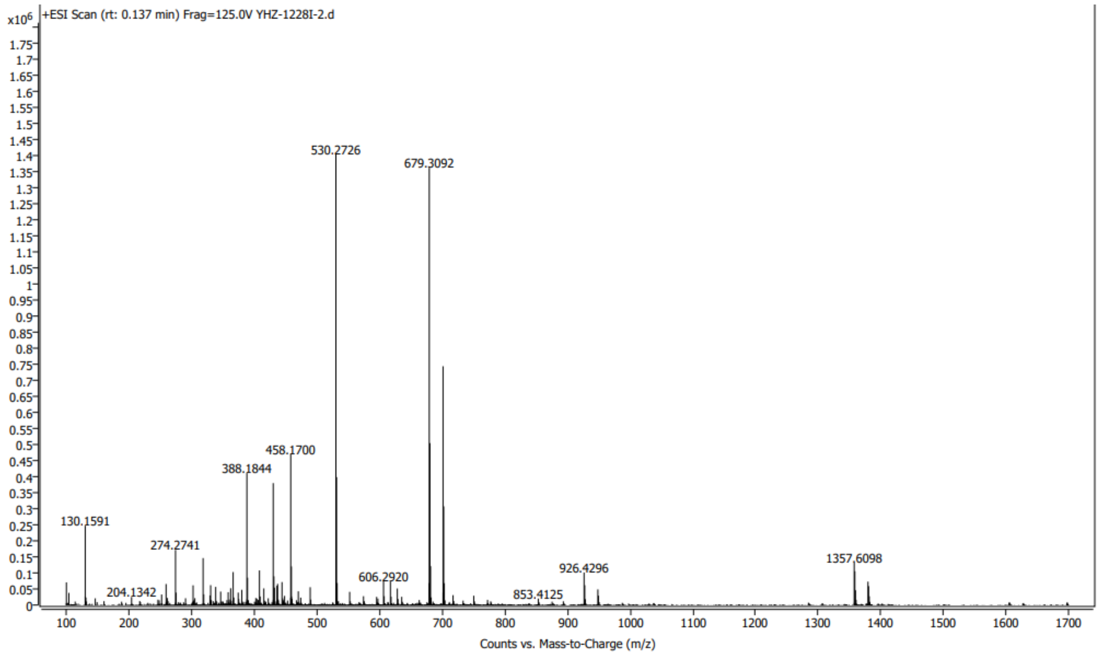
x10^6
+ESI Scan (rt: 0.137 min) Frag=125.0V YHZ-1228I-2.d
530.2726
679.3092
458.1700
388.1844
130.1591
274.2741
204.1342
606.2920
853.4125
926.4296
1357.6098
Counts vs. Mass-to-Charge (m/z)

MS of **I**

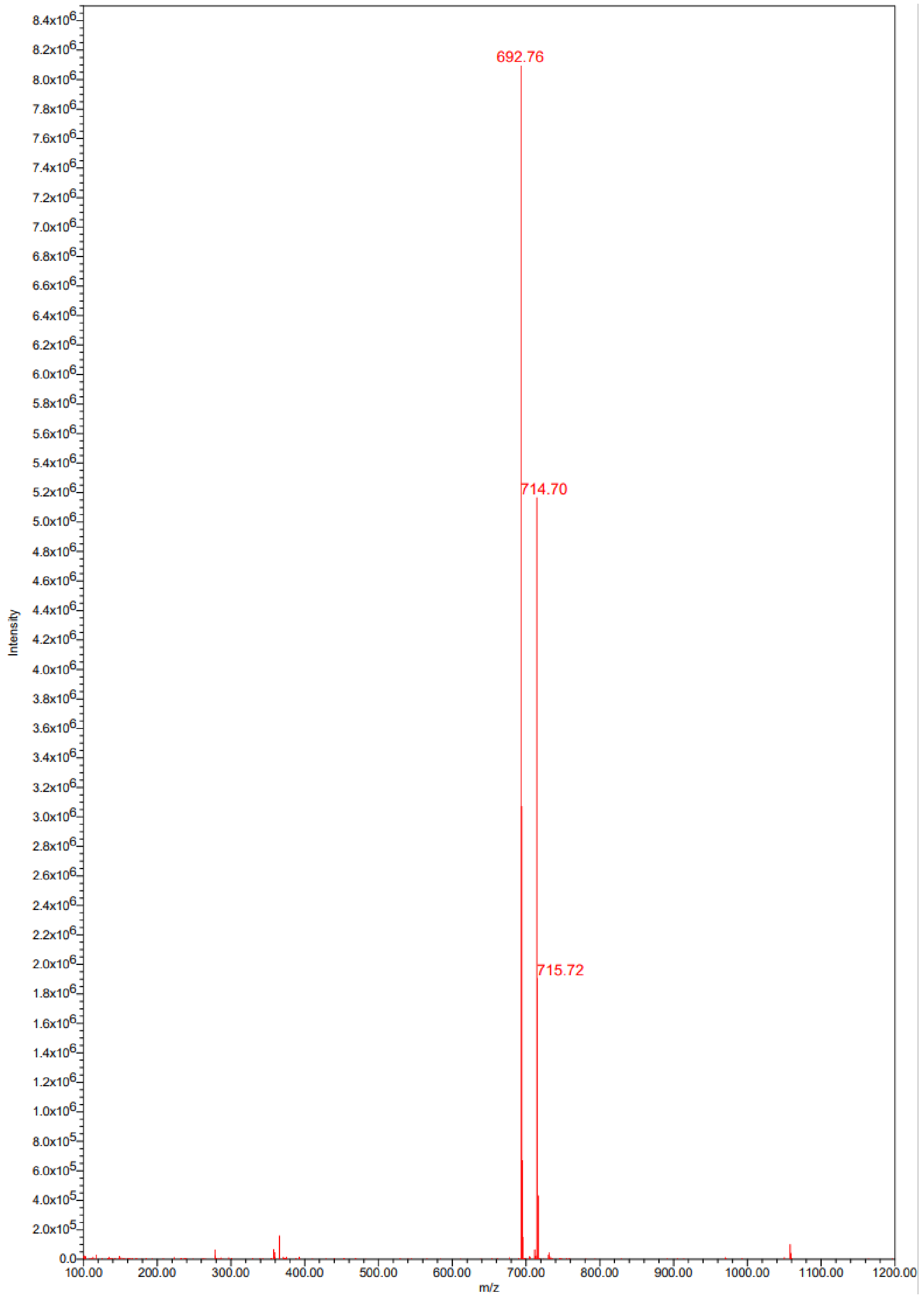

MS of **J**

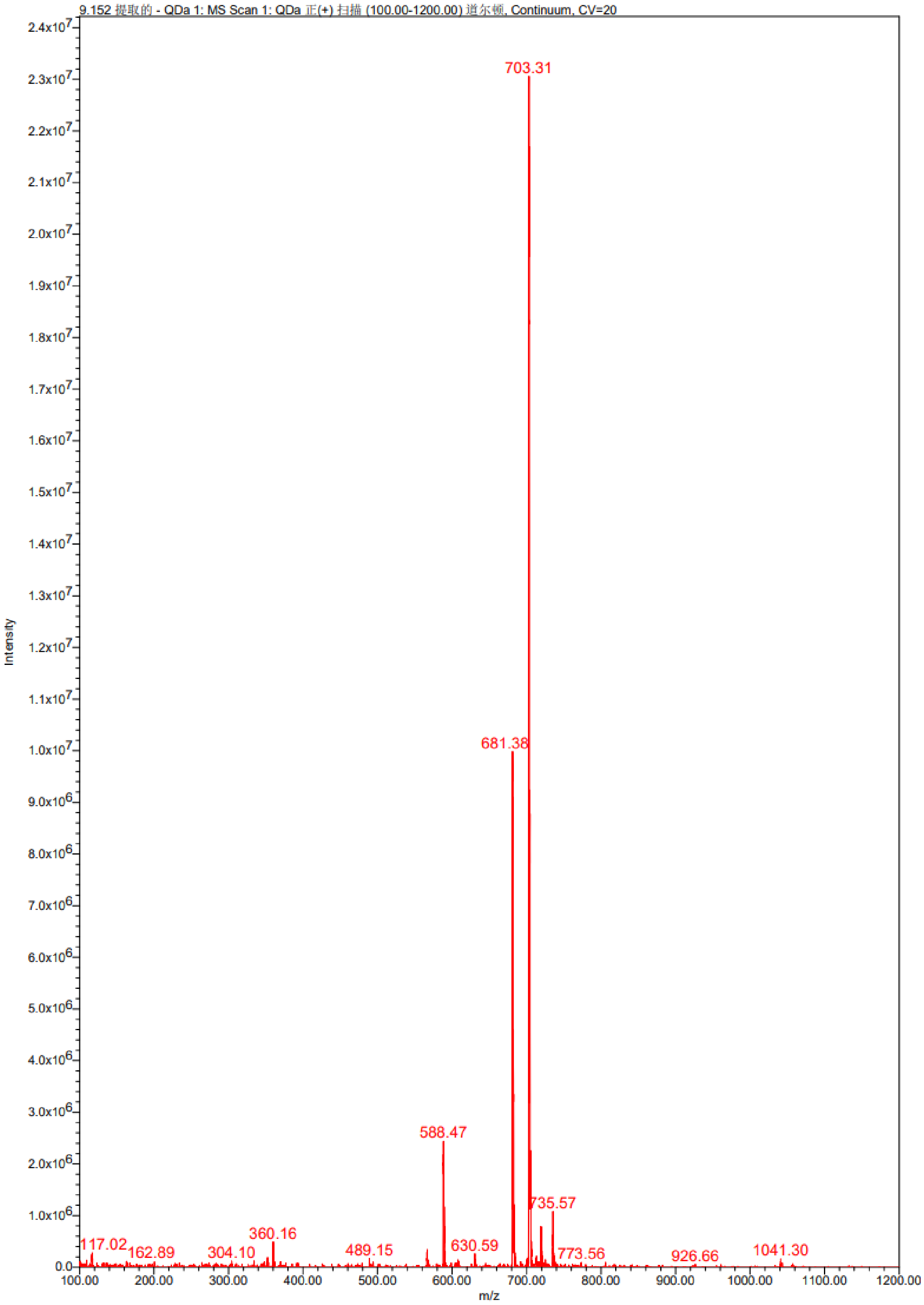
9.152 提取的 - QDa 1: MS Scan 1: QDa 正(+) 扫描 (100.00-1200.00) 道尔顿, Continuum, CV=20
Intensity
m/z
703.31
681.38
588.47
735.57
360.16
117.02
162.89
304.10
489.15
630.59
773.56
926.66
1041.30

MS of **K**

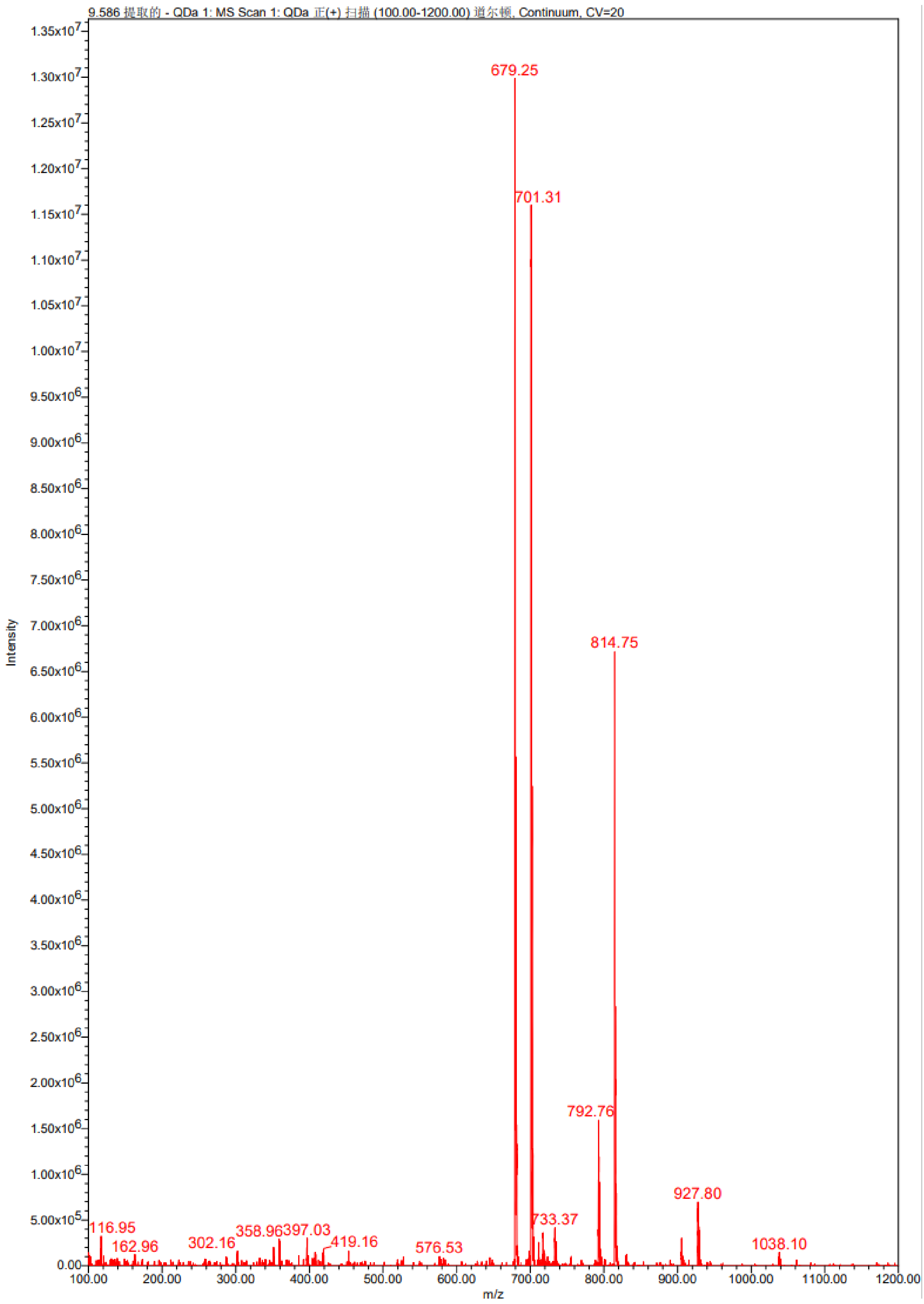
9.586 提取的 - QDa 1: MS Scan 1: QDa 正(+) 扫描 (100.00-1200.00) 道尔顿, Continuum, CV=20
Intensity
m/z
679.25
701.31
814.75
792.76
927.80
733.37
1038.10
116.95
162.96
302.16
358.96
397.03
419.16
576.53

MS of **L**

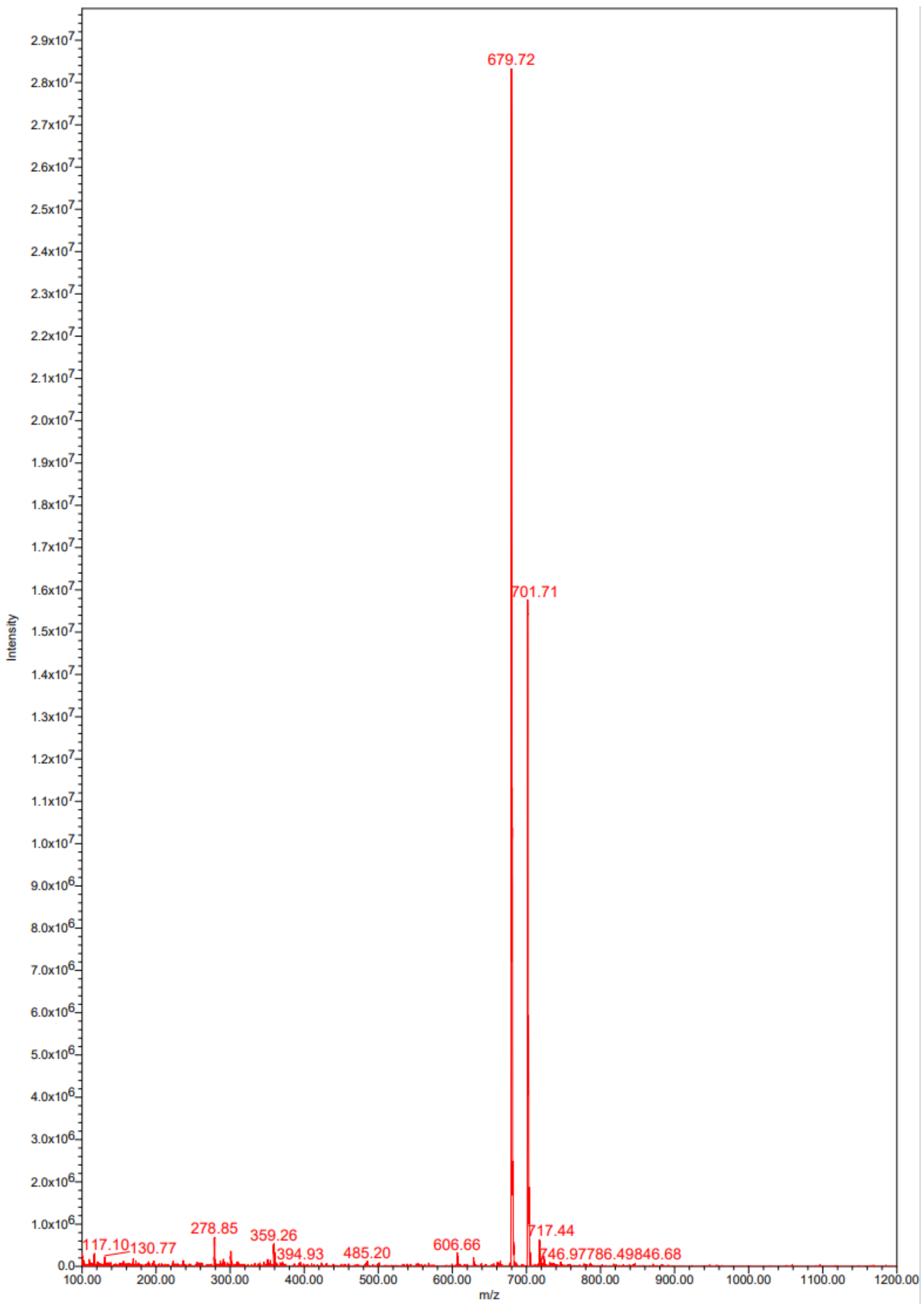
679.72
701.71
717.44
746.97786.49846.68
278.85
359.26
394.93
485.20
606.66
117.10130.77
Intensity
m/z
2.9x10^7
2.8x10^7
2.7x10^7
2.6x10^7
2.5x10^7
2.4x10^7
2.3x10^7
2.2x10^7
2.1x10^7
2.0x10^7
1.9x10^7
1.8x10^7
1.7x10^7
1.6x10^7
1.5x10^7
1.4x10^7
1.3x10^7
1.2x10^7
1.1x10^7
1.0x10^7
9.0x10^6
8.0x10^6
7.0x10^6
6.0x10^6
5.0x10^6
4.0x10^6
3.0x10^6
2.0x10^6
1.0x10^6
0.0
100.00
200.00
300.00
400.00
500.00
600.00
700.00
800.00
900.00
1000.00
1100.00
1200.00

MS of **M**

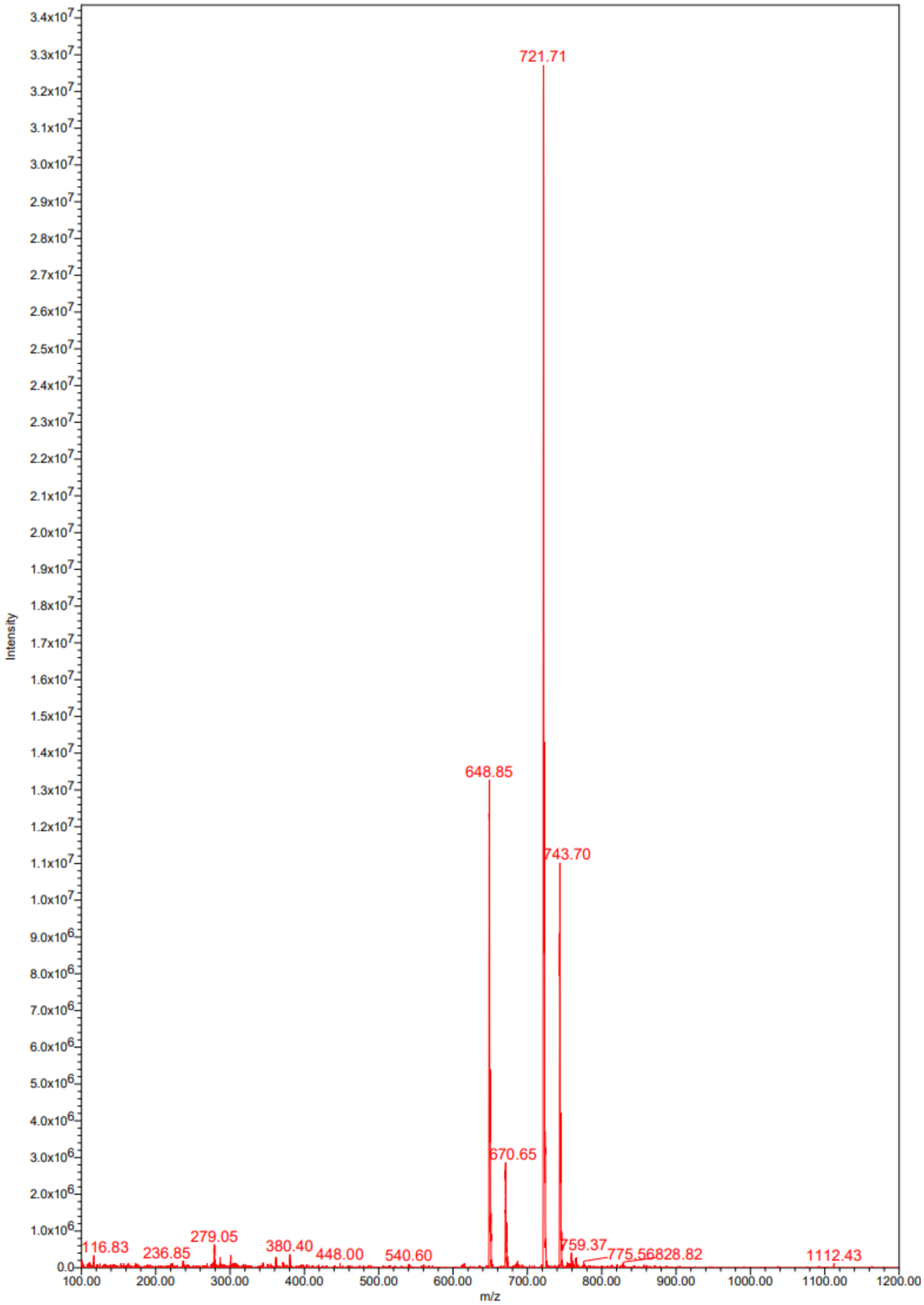

MS of **N**

4. Western Blotting

Figure S1. Western blotting of HDAC6/3/8 proteins in MDA-MB-231, HCT116

and MCF-7 cells treated with compounds A-N.

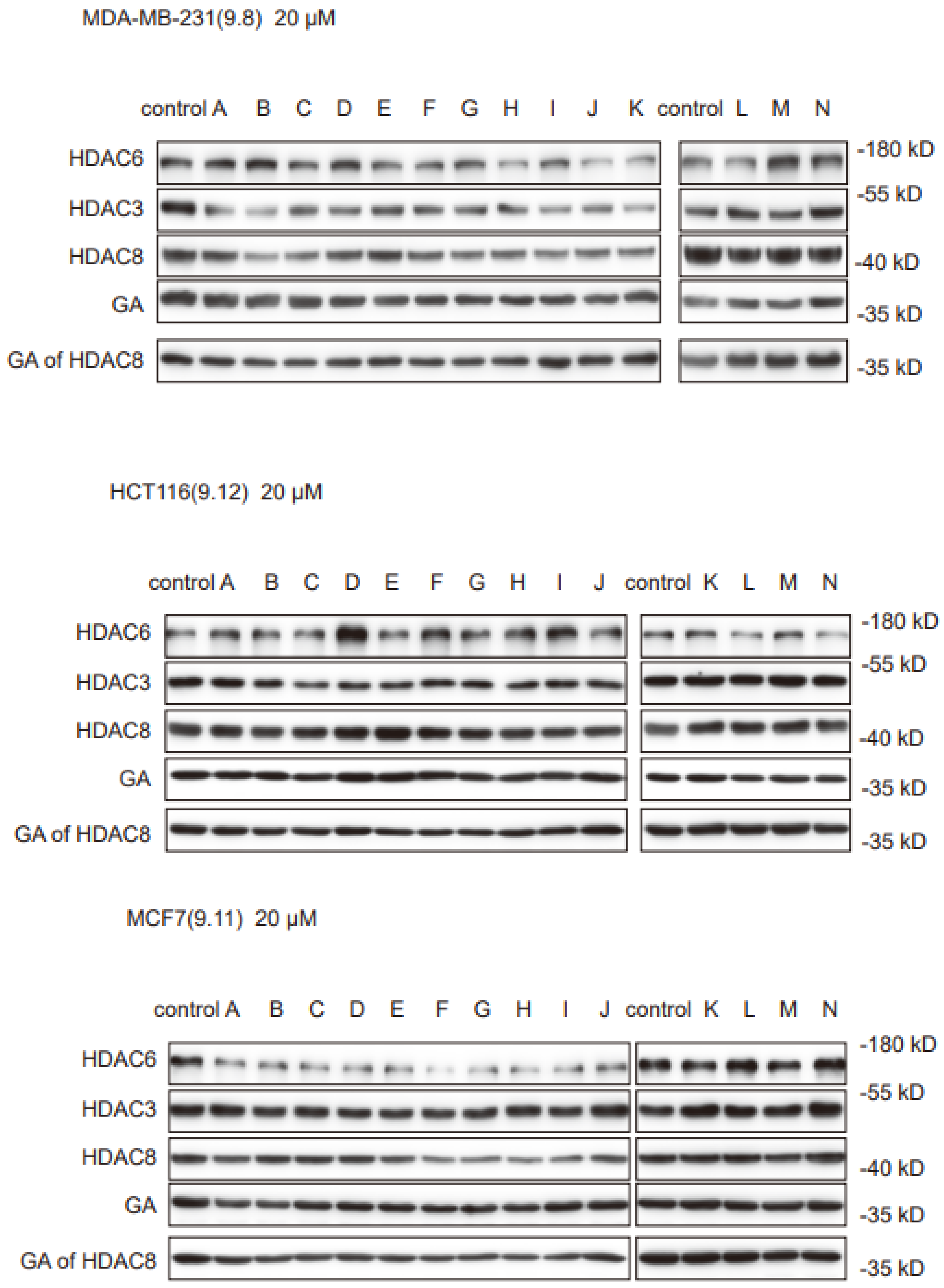

Figure S2. Western blotting of HDAC6/3/8 proteins in MCF-7 cells treated with compounds A-N. Representative blots of two independent biological replicates.

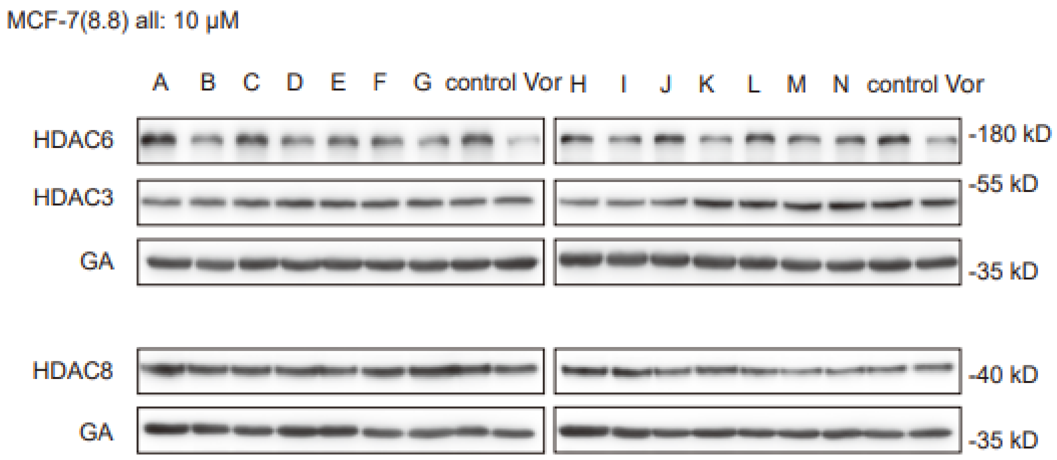

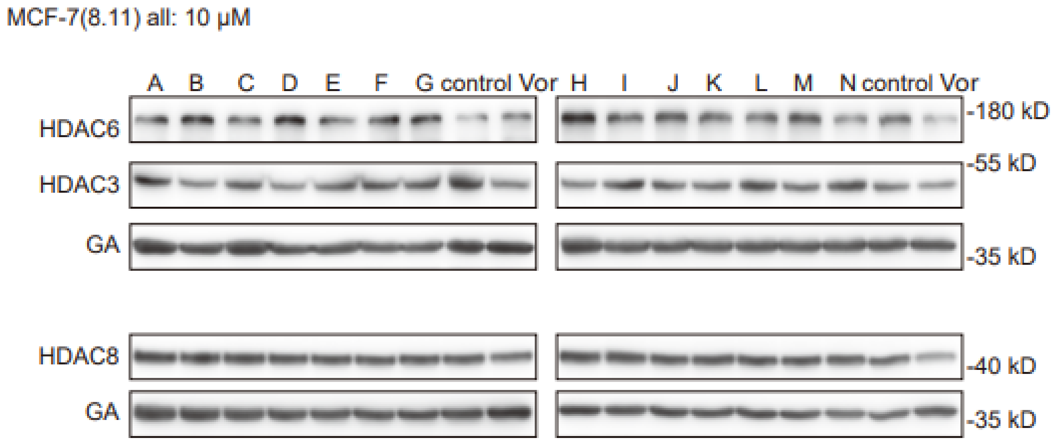

Figure S3. Western blotting of HDAC6/3/8 proteins in MDA-MB-231 cells treated with compounds A-N. Representative blots of two independent biological replicates.

MDA-MB-231(8.12) all: 10 μM

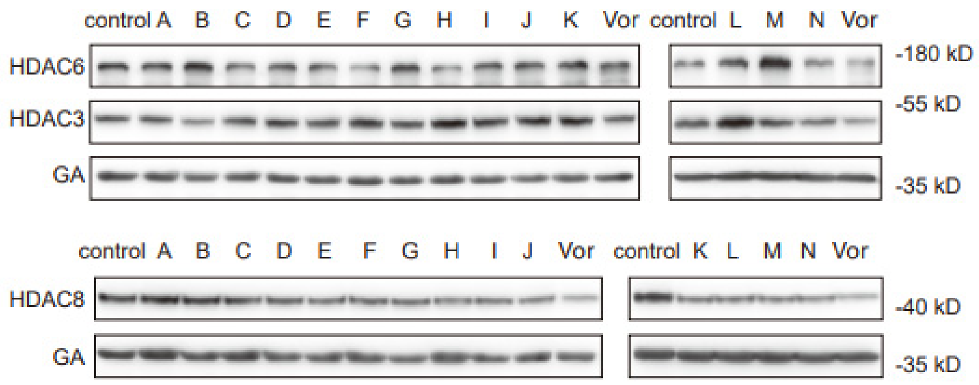

MDA-MB-231(8.15) all: 10 μM

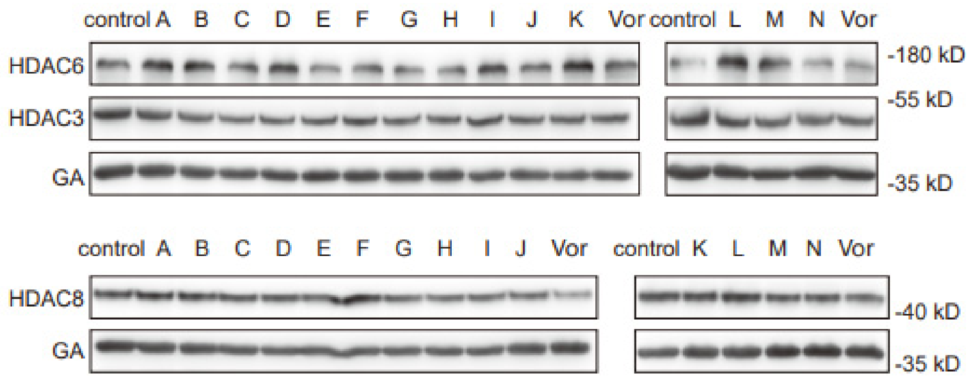

Supplement: Supplementary file 1 — Supplementary Material [file OPEN-15-e202500356-s001.pdf]
